# Transcription Factor TCF12‐Mediated Maternal Gene Expressions in Mouse Oocyte Are Prerequisites of Successful Fertilisation and Zygotic Genome Activation

**DOI:** 10.1111/cpr.70110

**Published:** 2025-08-03

**Authors:** Lan‐Rui Cao, Chi Zhang, Zuo‐Qi Deng, Yue‐xin Qiu, Zhao Zhang, Heng‐Yu Fan, Jing Li, Hong‐Bo Wu

**Affiliations:** ^1^ Zhejiang Key Laboratory of Precise Protection and Promotion of Fertility, Assisted Reproduction Unit, Department of Obstetrics and Gynecology, Sir Run Run Shaw Hospital, Life Sciences Institute Zhejiang University Hangzhou China; ^2^ Zhejiang University Medical School Hangzhou China; ^3^ State Key Laboratory of Reproductive Medicine Nanjing Medical University Nanjing China; ^4^ Department of Reproductive Medicine Qinzhou Maternity and Child Health Care Hospital Qinzhou China

**Keywords:** cell cycle, fertilisation, oocyte, preimplantation embryos, reproduction, transcription factor, zygotic genome activation

## Abstract

The maternal gene products stored in oocytes control the initial development of multicellular animals. Alteration within the dual allelic variants of transcription factor TCF12 causes female infertility; however, its impact on female reproduction is still unknown. In this study, we provide evidence that TCF12 is abundantly expressed within the nucleus of oocytes during growth at the germinal vesicle (GV) stage, recognising and binding to the functional domain of target genes to moderate transcriptional activity. The absence of *Tcf12* in oocytes during the primordial follicular phase causes female sterility. *Tcf12* does not participate in meiotic maturation; however, unlike *Tcf3*, it is essential for fertilisation and preimplantation development. *Tcf12* maintains fertilisation competence by controlling the proper expression and location of cortical granules and protease ovastacin (encoded by *Astl*). In contrast, zygotes without TCF12 have a prolonged mitotic cell cycle upon a decrease in protein phosphatase 2A (PP2A) activity inhibition, resulting in zygotic genome activation (ZGA) failure during the 2‐cell stage. Maternal knockout embryos gradually lose their developmental potential in subsequent developmental processes. These observations indicate that the maternal effect induced by *Tcf12* ensures preimplantation development.

## Graphical abstract

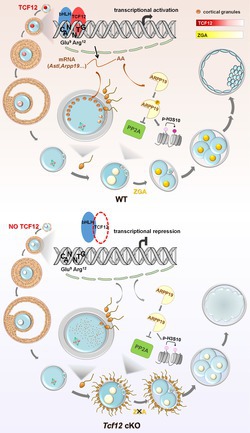

## 1 Introduction

The production of mature and fertilisation‐competent eggs is a lengthy and intricate process. From the embryonic stage onward, mammalian oocytes are halted during the first prophase of meiotic division [1]. After birth, the primary oocytes form primordial follicles enclosed by a single layer of flattened granulosa cells [2]. Dormant primordial follicles are activated and mature into primary, secondary, and antral follicles under the influence of follicle‐stimulating hormones (FSH) [3]. Subsequently, a sequence of events, including cumulus expansion, oocyte meiotic resumption, and release of the cumulus‐oocyte complex (COC), occurs under the influence of luteinizing hormone (LH) [4]. Meiotic maturation starts with germinal vesicle breakdown (GVBD) and concludes with the extrusion of the first polar body (PBE) [5, 6]. Oocytes remain in the second meiotic metaphase (MII) for many hours, waiting for the sperm to arrive [7].

During the growth of mammalian follicles, RNA polymerase‐dependent transcriptional activity is active in oocytes, massive maternal mRNA and protein increase exponentially, and the volume of the oocytes expands simultaneously [8, 9, 10]. As chromatin condenses into chromosomes, fully grown oocytes become transcriptionally silenced [11]. Subsequent development is ensured by the maternal products that accumulate during oocyte growth. Aberrant expression of maternal transcripts is directly linked to infertility as a result of oocyte growth arrest, compromising meiotic maturation and maternal‐to‐zygotic transition during the growth phase [12, 13]. For example, folliculogenesis‐specific basic helix–loop–helix α (FIGLA) and NOBOX oogenesis homeobox (NOBOX) are crucial in oogenesis and are implicated in the communication of cumulus‐oocyte complex [14, 15, 16, 17, 18]. bHLH transcription factor 1 (SOHLH1) and SOHLH2 play key roles in the formation, activation, and survival of primordial follicles during the development of gonads, spermatogenesis, and oogenesis [19, 20]. Maternal transcription factors could also prime ZGA, like heat shock transcription factor 1 (HSF1) and upstream stimulating factor 1 (USF1) [21]. The transcriptional activation of *Hsp90α* and minor ZGA gene *Hspa1b* by HSF1 is necessary for meiotic maturation and preimplantation embryo development, respectively [22, 23, 24]. USF1, a bHLH transcription factor, is a cis‐element of maternal genes essential for oocyte competence and early embryonic development up to the blastocyst stage [25]. Thus, transcription factors belonging to the bHLH family are important for female reproduction.

In mammals, E proteins are important derivatives of the bHLH transcription family, comprising TCF12 (also known as HEB), TCF3 (also known as E2A), and TCF4 (also known as E2‐2) [26]. E protein homodimers act as transcriptional activators. Heterodimers of E proteins with class II bHLH proteins recruit coactivators or co‐repressor complexes to access functions [27, 28]. A previous study indicated that TCF12 and TCF3 function as transcriptional coactivators of SMAD/FOXH1 during endodermal differentiation of human embryonic stem cells (ESCs) [29]. Moreover, TCF12 interacts with polycomb repressive complex 2 (PRC2) and SMAD2/3 to control cell fate in mouse ESCs [30]. Investigation of potential enhancer elements in mouse oocytes and nascent embryos revealed that TCF3/12 is a crucial regulator of follicular development. Analysis of transcriptome differences after the activation of primordial follicles revealed that *Tcf12* and *Tcf3* are highly expressed in oocytes during follicular development. However, depletion of *Tcf12* or *Tcf3* causes postnatal lethality, restricting the investigation of their functions in the female reproductive system [31, 32]. Moreover, oocyte‐specific ablation of *Tcf3/Tcf12* caused a dramatic decrease in primordial follicles, some of which developed into a primary follicle‐like form but did not advance to secondary follicles [33]. As a result, these oocyte‐selective *Tcf3/12*‐deleted female mice undergo premature ovarian failure at very young ages. Therefore, we could not analyse the function of TCF12 during oocyte‐to‐embryo transition in this mouse model.

Using conditional knockout mice as a model, we found that both TCF12 and TCF3 were required for the female reproductive system to regulate the expression of shared or unique targets. We provide evidence that TCF12 functions independently of TCF3 in female reproduction. Despite its involvement in oocyte maturation, TCF12 is crucial for fertilisation and preimplantation development.

## 2 Materials and Methods

### 2.1 Mice

The *Tcf12* ^*fl/fl*^;*Gdf9‐Cre* murine strain was established through targeted breeding between animals harbouring the well‐characterised *Tcf12* ^*fl*^ genetic variant and *Cre* recombinase‐expressing mice under *Gdf9* promoter regulation [32, 34]. Wild‐type (WT) mice were obtained from the Zhejiang Academy of Medical Science, China. All C57BL/6J mice were housed in a strictly regulated environment, featuring a consistent 12‐h light–dark cycle, maintained humidity levels ranging from 50% to 70%, and a temperature‐controlled atmosphere kept between 20°C and 22°C. The mice had continuous access to food and water. The feeding and experimental protocols for the mice adhered to national ethical standards and received approval from the Ethics Committee of Zhejiang University (approval code ZJU20210252).

### 2.2 Neonatal Cohort Evaluation

Each female mouse was cohabited with one proven fertile male of reproductive age to control for paternal variables. Mating success was systematically verified through twice‐daily monitoring for vaginal plug formation, with initial observation timing establishing gestational reference point E0.5. Neonatal cohorts were harvested at parturition (P0 timepoint) and subjected to comprehensive biometric processing within a 24‐h window, including enumeration and mass measurement protocols to establish both quantitative brood parameters and neonatal survival metrics. The implemented standardisation process reduces experimental fluctuations while maintaining consistent assessment of maternal reproductive performance.

### 2.3 Harvesting Oocytes and In Vitro Culture

Female mice aged 23–26 days received an injection of 5 IU pregnant mare serum gonadotropin (PMSG, Ningbo Sansheng Pharmaceutical, veterinary drugs 110,044,564) and were euthanised 44 h post‐injection. Fully grown GV stage oocytes were collected in M2 medium (Sigma‐Aldrich, M7167) and cultured in M16 medium (Sigma‐Aldrich, M7292) in mineral oil (Sigma‐Aldrich, M8410) at 37°C in a 5% CO_2_ atmosphere [6].

### 2.4 Superovulation and Natural Fertilisation

Female mice aged 23–26 days received an injection containing 5 IU of human chorionic gonadotropin (hCG), sourced from Ningbo Sansheng Pharmaceutical and registered under veterinary drug number 110041282, approximately 48 h after a 5 IU PMSG injection. Following 16 h of hCG administration, COCs were collected from the oviducts of female mice. COCs were treated with hyaluronidase to remove cumulus cells, yielding mature metaphase II (MII) oocytes. Female mice were immediately paired with adult males post‐hCG injection for in vivo fertilisation. Successful fertilisation was confirmed by the presence of a vaginal plug the following morning. Zygotes were collected from fallopian tubes 22 h post‐hCG injection; 0 h was designated as the time point of successful fertilisation. Embryos derived from natural mating were categorised by developmental stage: 1‐cell (zygote, 0–24 h), 2‐cell (24–36 h), 4‐cell (36–48 h), 8‐cell (48–72 h), morula (72–96 h), and blastocyst (96–144 h). Embryos were collected at designated intervals post‐coitus for subsequent analysis.

### 2.5 In Vitro Fertilisation and Parthenogenetic Activation

Oocytes at the MII stage were used for in vitro fertilisation (IVF) or parthenogenetic activation (PA). Fresh capacitated semen was diluted in IVF medium for 1 h for IVF. Then, oocytes harvested from five mice were placed in 200 μL droplets of IVF medium. A semen suspension (3–5 μL) was added to the oocyte droplets, diluting the concentration to 4 × 10^6^ sperm cells/mL, and co‐incubated for 4–6 h. For PA, oocytes were initially incubated in a calcium‐free activation medium for 3 h before being transferred to a calcium activation medium lacking strontium chloride. Following IVF or PA, zygotes were cultured in KSOM (Millipore, MR‐106‐D) to promote further development.

### 2.6 Live‐Cell Imaging

Parthenogenetic embryos were cultured in a calcium activation medium after 6 h of PA for live‐cell imaging. Images were acquired using a BioTek Invented Living Cell Workstation. Image acquisition was performed every 30 min using Gen5 software.

### 2.7 Sperm‐Binding Assay

Capacitation of sperm harvested from adult WT mice was performed in IVF medium for 1 h. Capacitated sperm were co‐incubated with ovulated eggs or two‐cell embryos for 30 min. WT 2‐cell embryos served as positive controls, and WT eggs were used as negative controls.

### 2.8 In Vitro Transcription and mRNA Microinjection

The expression vectors were linearised using specific restriction enzymes to prepare mRNAs for microinjection. In vitro transcription of 5′‐capped mRNAs was performed using the T7 or SP6 message mMACHINE Kit (Invitrogen, AM1344 or AM1340) for 4 h at 37°C. Transcribed mRNAs were polyadenylated using a Poly (A) Tailing Kit (Invitrogen, AM1350), purified using lithium chloride precipitation, and finally dissolved in nuclease‐free water. Microinjection was performed using a microinjector and micromanipulator under a microscope (Eclipse TE200, Nikon). Zygotes were injected with 5–10 pL of mRNA samples, diluted to 200 μg/mL for synthetic mRNA or 20 μM for siRNA.

### 2.9 Immunofluorescence

Oocytes and embryos were subjected to a fixing process involving a 4% paraformaldehyde solution, prepared in phosphate‐buffered saline (PBS) for 20–30 min, followed by permeabilisation in PBS with 0.3% Triton X‐100 for 20–30 min. Subsequently, samples were blocked with PBS containing 1% bovine serum albumin for 30 min, followed by incubation with primary and secondary antibodies at 25°C for 1 h each. Nuclei were stained with 4′,6‐diamidino‐2‐phenylindole (DAPI) for 15–30 min. The images were acquired using a Zeiss LSM710 confocal microscope. Antibodies used in this study are listed in Table S1. Z‐stack images were captured using a confocal microscope with identical exposure/gain settings across samples to ensure comparability for targeting the staining and quantitative analysis of ASTL and cortical granules in oocytes. A 2‐μm‐wide band at the cell periphery was defined using the “ROI Manager” tool in ImageJ (v1.53) to define the cortical region, and intracellular areas > 5 μm from both the nucleus (DAPI‐defined) and the cortical band were selected as a cytoplasmic region. Thereafter, background fluorescence (from cell‐free regions) was subtracted. Mean intensity values for cortical and cytoplasmic regions were calculated using the “Measure Stack” plugin. The cortical/cytoplasmic (C/CC) ratio was normalised to the cell‐wide mean intensity.

### 2.10 Detection of Transcription and DNA Replication

Transcriptional activity was ascertained by incubating oocytes or embryos in media containing 1 mM of 5‐ethynyl uridine (EU) for 2 h, followed by staining using the Click‐iT RNA Alexa Fluor 488 Imaging Kit, supplied by Life Technologies (product code C10329). Zygotes at various pronuclear stages were incubated in KSOM medium containing 10 μM of 5‐ethynyl‐2′‐deoxy‐uridine (EdU) for 2 h and stained with a Click‐iT EdU Alexa Fluor 488 Imaging Kit (Life Technologies, C10337) to detect DNA replication. The EU assay detects nascent RNA synthesis by incorporating nucleotide analogs during transcription. Incorporated EU is conjugated with fluorescent azide via click chemistry, allowing quantitative visualisation of transcriptional activity in embryos. EdU was introduced into the culture medium of mouse zygotes approximately 2 h prior to reaching the predetermined pronuclear formation stage. This thymidine analog was dissolved in pre‐equilibrated embryo culture medium at a final concentration of 10 μM, ensuring optimal bioavailability while maintaining embryo viability. The timing of administration was carefully synchronised with embryonic developmental progression, calculated from the observed fertilisation time (designated as 0 h post‐fertilisation). During this critical pre‐pronuclear phase, actively dividing zygotes incorporate EdU into newly synthesised DNA strands during the S‐phase of their cell cycle. The 2‐h pretreatment window was established based on previous kinetic studies of DNA replication patterns in early murine embryogenesis, allowing sufficient time for thymidine analog incorporation while avoiding interference with pronuclear membrane formation. Following EdU exposure, zygotes were thoroughly washed through three sequential droplets of fresh culture medium to remove residual compounds. Subsequent incubation continued under standard embryo culture conditions (37°C, 5% CO₂, humidified atmosphere) until the target pronuclear developmental stage was reached. This temporal administration protocol enables precise labeling of DNA synthesis events preceding pronuclear maturation, facilitating later detection of replication‐active nuclei through click chemistry‐based visualisation methods. The concentration and duration were optimised to achieve robust nuclear labeling while maintaining developmental competence, as validated by control experiments measuring blastocyst formation rates. This approach provides temporal resolution for studying DNA replication dynamics during the crucial zygote‐to‐embryo transition phase. The mean nuclear signal was acquired and processed using a Zeiss LSM710 confocal microscope and the ImageJ software.

### 2.11 Histological Analysis

Ovaries were gathered and preserved in PBS containing 10% formaldehyde at 4°C for 18 h. Ovaries were dehydrated, paraffin‐embedded following standard protocols [35, 36], sectioned at 5 μm thickness, and stained with haematoxylin and eosin. Immunohistochemistry (IHC) was conducted utilising the ABC kit (Vectastain, PK‐6100) and the 3,3′‐diaminobenzidine peroxidase substrate kit (Vectastain, SK‐4100) with the antibodies specified in Table S1.

### 2.12 Extraction of Minute Quantities of RNA, Reverse Transcription (RT), and RT‐PCR

Ten collected cells were lysed in a lysis buffer containing Triton X‐100 and RNase inhibitor. After RNA isolation, RT was performed using the SuperScript II Reverse Transcriptase. The PCR products were diluted 6–9 times with nuclease‐free water and used as templates for RT‐PCR. Quantitative and semi‐quantitative RT‐PCR were conducted using the ABI 7500 Real‐Time PCR system with the primers specified in Table S2. Relative mRNA levels were calculated using Microsoft Excel and normalised using endogenous Actin mRNA as an internal control. The qPCR reactions were conducted in triplicate or quadruplicate. The primer sequences are listed in Table S2.

### 2.13 Western Blot Analysis

Protein samples were lysed in a cracking buffer with β‐mercaptoethanol and then heated at 95°C for 10 min. Total protein was separated using SDS‐PAGE and electrophoresed onto PVDF membranes (Millipore, Bedford, MA, USA). After blocking the membranes with 5% non‐fat milk for 30 min, primary antibodies were probed for 16 h at 4°C. Membranes were incubated with a secondary antibody (Jackson ImmunoResearch Laboratories) for 1 h after three washes. After three additional Tris‐buffered saline with Tween (TBST) washes, the signals were detected using Super Signal West Femto Maximum Sensitivity Substrate (Thermo Fisher Scientific, Waltham, MA, USA). Table S1 lists the primary antibodies used and their dilutions.

### 2.14 RNA‐Sequencing and Data Analyses

Oocytes at the GV stage were collected from female mice aged 14.5 days. Fully grown oocytes at the GV stage were harvested from 23 to 26‐day‐old female mice 44–48 h post‐PMSG injection. Considering the cell cycle delay in knockout embryos, late 2‐cell embryos of wild‐type controls were collected at 26–28 h post‐fertilisation, while knockout embryos were systematically collected at 30–32 h post‐fertilisation. Each sample, consisting of 10 oocytes or embryos, was treated with 4 μL of lysis buffer containing oligo‐dT primers, dNTPs, 0.2% TritonX‐100 and an RNase inhibitor, and immediately converted to cDNA using the Smart‐seq2 method as previously described [37]. The sequencing libraries were constructed using the TruePrep DNA Library Prep Kit V2 for Illumina (Vazyme, TD503). The sequencing output was aligned to the mm9 reference genome using Bowtie2 and TopHat2 software (version 2.0.14). These tools are engineered to allow accurate mapping across splice boundaries by breaking down the sequencing reads into segments. Using Cuf‐flinks version 2.2.1 with the University of California Santa Cruz (UCSC) gene model reference annotation, 77% of unique reads were assembled into transcripts. Gene expression levels were quantified using normalised as fragments per kilobase of transcript per million mapped reads (FPKM). Genes exhibiting differential expression were identified by asking for a fold‐change ≥ 2 with FPKM ≥ 2 in at least one sample.

### 2.15 Mass Spectrometry Sample Preparation and Data Analyses

Fully grown GV‐stage oocytes were harvested from 23 to 26‐day‐old female mice 44–48 h post‐PMSG injection. The oocytes were briefly treated with Tyrode's acid solution (Sigma‐Aldrich) to eliminate the zona pellucida. The oocytes were collected into 0.2‐mL RNA‐free centrifuge tubes following three washes in 0.3 mol/L sucrose solution. Samples containing 100 oocytes were prepared for MS using in‐solution digestion. Samples were loaded onto an analytical column and analysed with gradient at a flow rate of 300 nL/min for 60 min using the Easy‐nLC 1200 system. The Q Exactive HF‐X mass spectrometer was operated in data‐dependent mode. Mass spectrometry analysis was performed using MaxQuant (version 1.6.10.43). Proteins exhibiting a fold‐change > 2 in at least one sample were identified as differentially expressed.

### 2.16 Statistical Analysis

The results are reported as the arithmetic mean accompanied by the standard error of measurement (SEM). Each experimental procedure was repeated at least three times to ensure reproducibility. We utilised two‐tailed unpaired Student's *t*‐tests to facilitate comparisons between the two experimental sets. ‘n.s’ denotes non‐significance. The significance levels were denoted as follows: *p < 0.05, **p < 0.01, and ***p < 0.001.

## 3 Results

### 3.1 TCF12 Is a Maternal Transcription Factor Located Specifically in the Nucleus of Growing GV Oocytes

**FIGURE 1:**
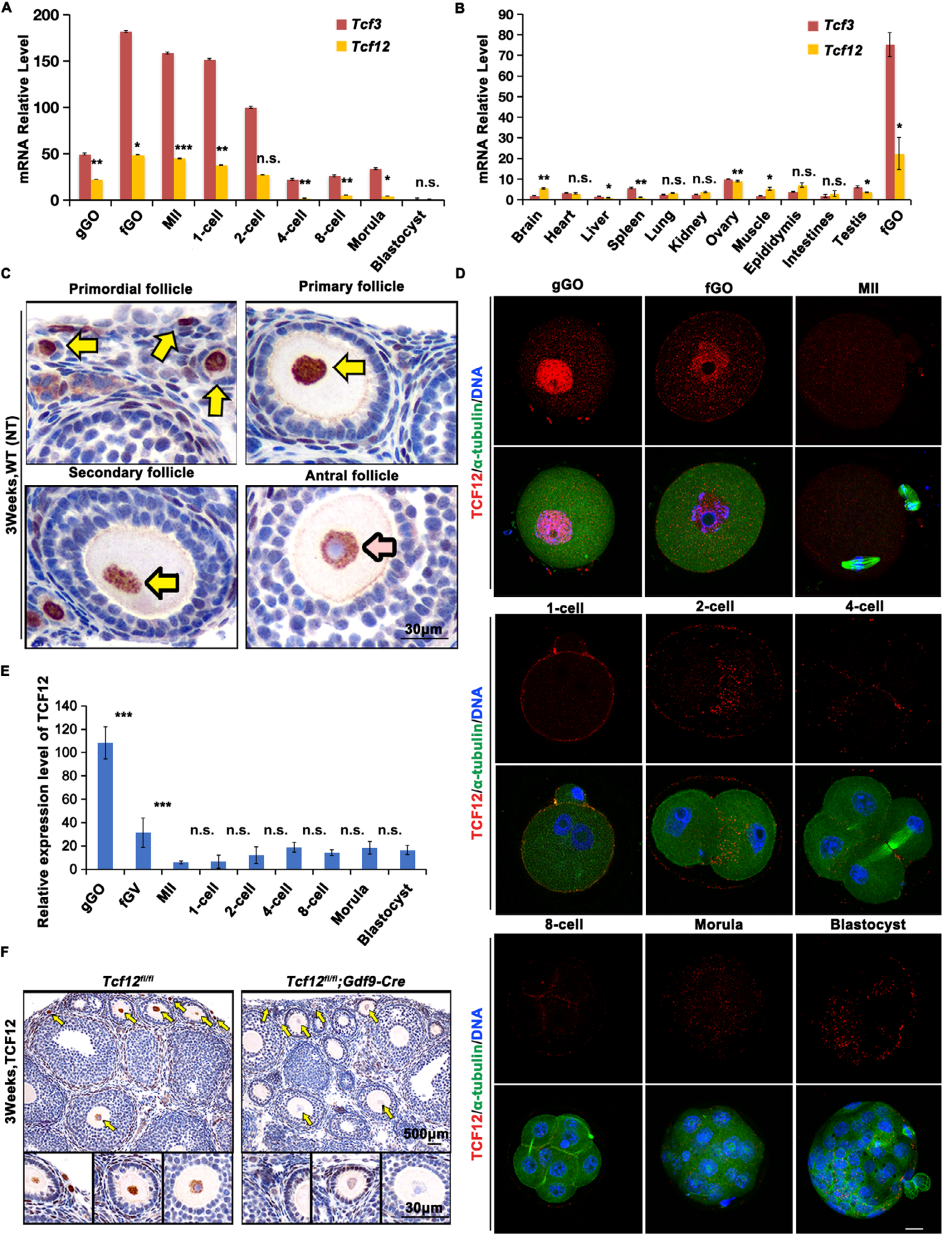
Expression pattern of TCF12 in mouse oocytes and preimplantation embryos. (A, B) Relative mRNA levels of *Tcf12* and *Tcf3* in oocytes, preimplantation embryos, and various somatic tissue samples measured using quantitative RT‐PCR (qRT‐PCR). gGO: Growing GV oocyte; fGO: Fully‐grown GV oocyte. *n* = 4 biological replicates. Error bars, SEM; n.s., non‐significant; *p < 0.05; **p < 0.01; ***p < 0.001, computed using two‐tailed Student's *t*‐tests. (C) Immunohistochemical (IHC) staining on 3‐week‐old wild‐type ovarian sections showing the expression of TCF12 in indicated follicular developmental stages. The yellow arrows indicate oocytes in primordial, primary, and secondary follicles. The pink arrow indicates oocytes in pre‐ovulatory follicles. Scale bar, 30 μm. (D) Immunofluorescence (IF) of TCF12 (red) and α‐tubulin (green) in oocytes and preimplantation embryos. DNA was dyed blue with 4′6‐diamidino‐2‐phenylindole (DAPI). More than 25 oocytes or embryos were analysed at each stage. Scale bar, 25 μm. (E) The dynamic change of TCF12 during mouse oocytes and early‐embryonic development. The fluorescence intensity of TCF12 of each sample was obtained using the ‘Measure’ function of ImageJ and the percentage of maximal fluorescence intensity was presented; fluorescence intensity was quantitatively analysed in 10 cells per time point. (F) IHC results displaying the expression of TCF12 in oocytes of mice with indicated genotypes. The nuclei of oocytes are indicated using yellow arrows. Scale bars, 500 and 30 μm.

The specific expression profiles of *Tcf12* and *Tcf3* were assessed using quantitative RT‐PCR. *Tcf12* and *Tcf3* transcripts were highly expressed in oocytes compared to that in tissues or preimplantation embryos (Figure 1A,B). Immunohistochemical (IHC) staining showed that TCF12 was exclusively localised to the nuclei of oocytes at different phases of follicle development (Figure 1C, arrows). The highest expression levels of TCF12 were observed at the primordial and primary follicle stages and then declined with follicle development (Figure 1C, pink arrow). Immunofluorescence (IF) results further proved that the TCF12 protein was highly expressed in the nucleus of growing oocytes, decreased following meiotic resumption, and became undetectable throughout preimplantation embryo development (Figure 1D,E). We crossed *Tcf12* ^*fl/fl*^ mice with *Gdf9‐Cre* mice to specifically delete *Tcf12* during the primordial follicle stage to investigate the physiological functions of TCF12. Knockdown efficiency was confirmed using IHC, and TCF12 was removed from the secondary follicle stage onward (Figure 1F).

### 3.2 TCF12 Is Crucial for Fertilisation and Early Embryonic Development in Mice

**FIGURE 2:**
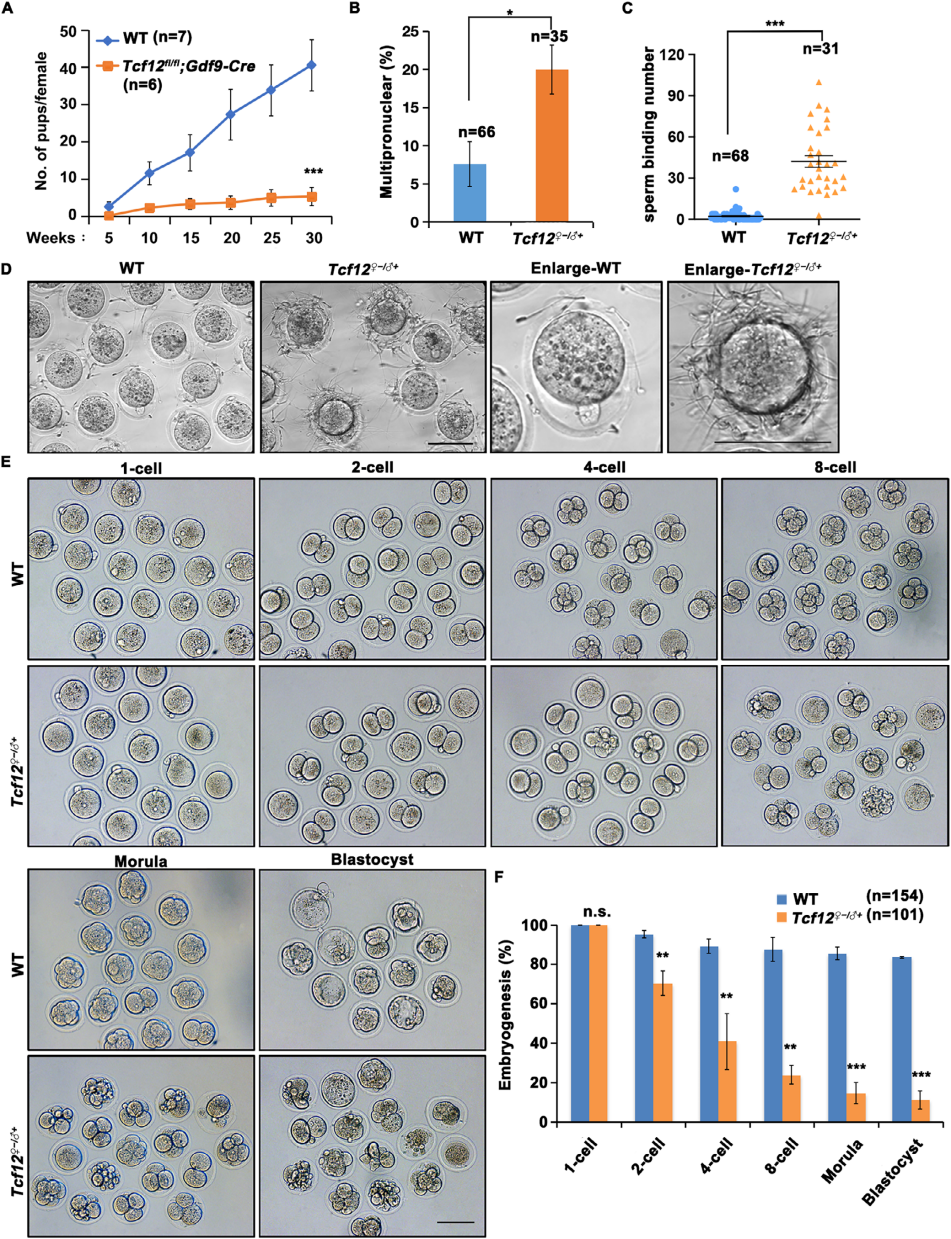
TCF12 is crucial for fertilisation and early embryonic development in mice. (A) Fertility test of WT (*n* = 7) and *Tcf12* ^*fl/fl*^; *Gdf9‐Cre* (*n* = 6) female mice. (B) Percentage of the multi‐pronuclear zygotes at 8 h after in vitro fertilisation (IVF). The WT and *Tcf12* ^*fl/fl*^;*Gdf9‐Cre* eggs were in vitro fertilised with capacitated sperms of WT males for 6 h. Total numbers of zygotes used are indicated (*n*). (C) Numbers of sperms in the perivitelline space at 8 h after IVF. (D) Representative images using time‐lapse imaging of the zygotes with indicated genotypes at 8 h after IVF. Scale bar, 100 μm. (E) Representative images of the in vivo fertilised embryos that derived from the oviducts of WT and *Tcf12* ^*fl/fl*^;*Gdf9‐Cre* female mice at the indicated stages. Scale bar, 100 μm. (F) Percentage of the in vivo fertilised embryos that derived from WT and *Tcf12* ^*fl/fl*^;*Gdf9‐Cre* female mice and reached the corresponding stages. Total numbers of analysed embryos are indicated (*n*). Error bars, SEM; n.s., non‐significant; *p < 0.05; **p < 0.01; ***p < 0.001, computed using two‐tailed Student's *t*‐tests.

*Tcf12* ^*fl/fl*^;*Gdf9‐Cre* females and their control littermates were crossed with adult wild‐type (WT) males for 7 months to evaluate the effect of the knockout *Tcf12* on fertility. Only a few pups were born to *Tcf12* ^*fl/fl*^;*Gdf9‐Cre* females; that is, *Tcf12* ^*fl/fl*^;*Gdf9‐Cre* females had strong sub‐fertility (Figure 2A). The absence of *Tcf12* did not affect follicle maintenance or growth (Figure S1A). In addition, the intrinsic ovarian response after superovulation treatment remained intact (Figure S1B,C). Fully grown GV oocytes isolated from PMSG‐treated *Tcf12* ^*fl/fl*^;*Gdf9‐Cre* females underwent normal spontaneous in vitro maturation (Figure S1D,E). Therefore, TCF12 is not essential for oocyte growth or meiotic maturation under physiological conditions. Although *Tcf12* ^*fl/fl*^;*Gdf9‐Cre* MII‐stage oocytes appeared normal, they could not be fertilised regularly. The polyspermy rate increased as *Tcf12* ^*fl/fl*^;*Gdf9‐Cre* MII oocytes underwent in vitro fertilisation treatment (Figure 2B). Meanwhile, an elevated occurrence of extra spermatozoa within the perivitelline space was noted in maternal *Tcf12* knockout zygotes (*Tcf12* ^*♀−/♂+*^) during the experiment (Figure 2C,D). Next, we mated superovulated females (*Tcf12* ^*fl/fl*^;*Gdf9‐Cre* and *Tcf12* ^*fl/fl*^) with WT males and obtained zygotes from fallopian tubes that were cultured in vitro. In contrast to the control group, knockout embryos arrested to some extent at every stage, and only very few embryos could develop to the blastocyst stage (Figure 2E,F). These results indicated that the maternal transcription factor TCF12 is crucial for fertilisation and early embryonic development in mice.

### 3.3 TCF12 Is Required to Regulate the Accumulation of Maternal mRNA During Oocyte Growth

**FIGURE 3:**
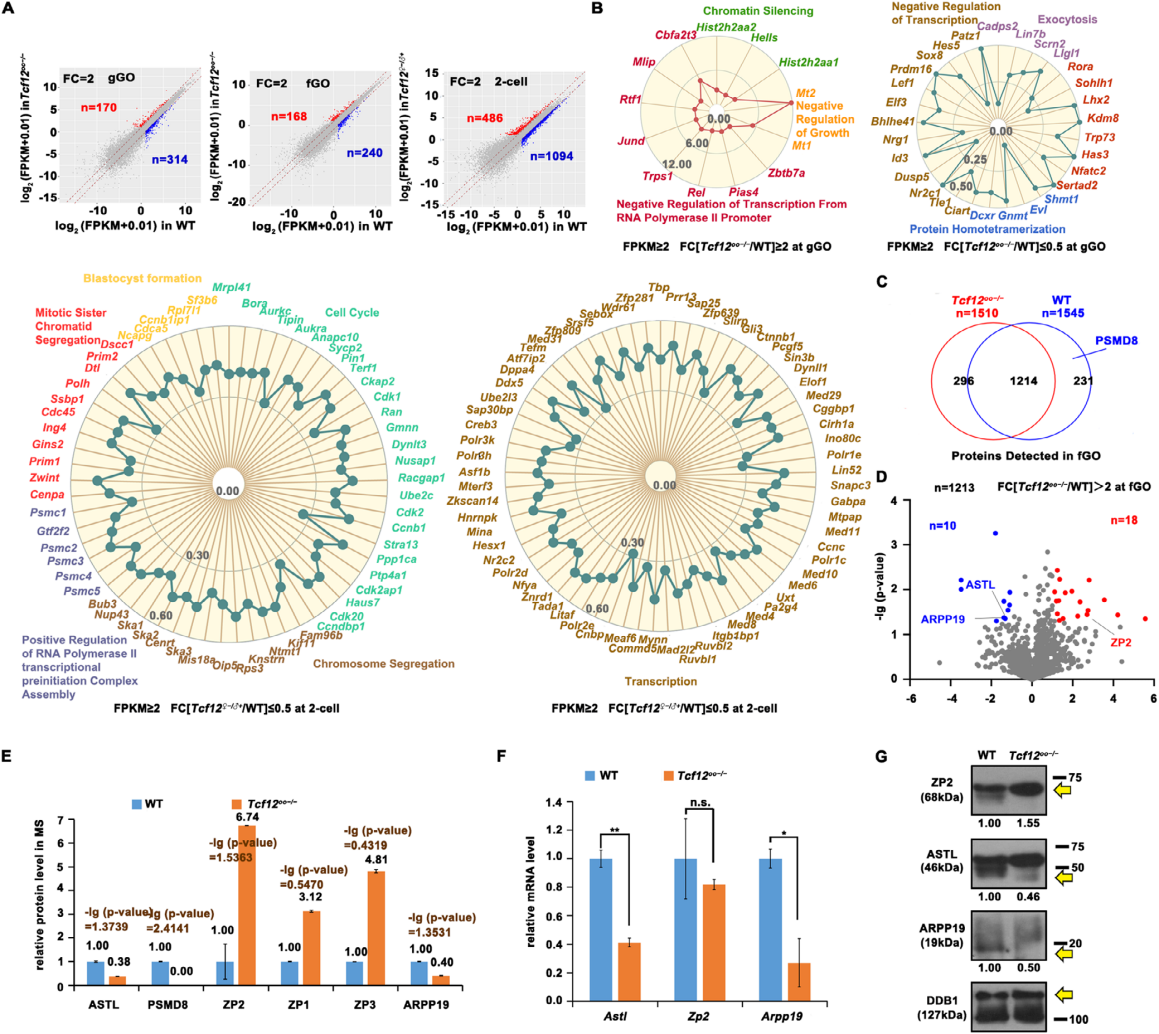
RNA‐sequencing and protein mass spectrometry analyses of *Tcf12* null oocytes. (A) Scatter plot comparing the transcriptomes of growing GV oocytes (gGV), fully grown GV oocytes (fGV), and 2‐cell embryos derived from WT and *Tcf12* ^*fl/fl*^;*Gdf9‐Cre* females. Transcripts that were downregulated more than 2‐fold in *Tcf12*‐deleted samples are highlighted in red (up) and blue (down). (B) Gene ontology (GO) analysis of differentially expressed transcripts at growing GV and 2‐cell stage in *Tcf12* ^*fl/fl*^;*Gdf9‐Cre* females compared with those in the control. Numbers represent the ratios of mRNA levels over control. (C) Venn diagram of proteins which are detected in WT and *Tcf12* ^*fl/fl*^;*Gdf9‐Cre* fully grown GV oocytes. (D) Scatter dot plot indicating the Log_2_ (FC in expression values) vs. −lg (p value) of differentially expressed proteins at the fully grown GV stage in *Tcf12* ^*fl/fl*^;*Gdf9‐Cre* females compared with those in the control. Proteins that varied more than 2‐fold in *Tcf12*‐deleted oocytes are highlighted in red (up) or blue (down). (E) Relative level of phenotype‐associated proteins detected in LC–MS/MS analysis. (F) Relative mRNA levels of phenotype‐associated genes measured using qRT‐PCR. *n* = 3 biological replicates. (G) Western blot analysis of ASTL, ZP2, and ARPP19 in fully grown GV oocytes derived from WT and *Tcf12* ^*fl/fl*^;*Gdf9‐Cre* females. DDB1 was used as the loading control. Numbers below blots represent the relative band intensities measured using ImageJ. In each lane, total protein from 150 oocytes was loaded. Given the high background observed with the ASTL antibody, the yellow arrow indicates the anticipated ASTL band.

We performed RNA‐seq on *Tcf12* ^*fl/fl*^ and *Tcf12* ^*fl/fl*^;*Gdf9‐Cre* growing GV oocytes (gGO), fully grown GV oocytes (fGO), and embryos at the 2‐cell stage to better understand the role of *Tcf12* in the accumulation of maternal transcripts at the molecular level. The gene expression was calculated and reported as FPKM (Table S5). A significant correlation was found between the replicates in each independent group (Figure S2A, Tables S3 and S4). The Spearman correlation coefficients and quality control among WT and *Tcf12* ^*oo−/−*^ oocytes or *Tcf12* ^*♀−/♂+*^ embryos are listed separately in Tables S3 and S4. The deletion of *Tcf12* did not affect the total mRNA levels in each period (Figure S2B). There was a great contrast between the *Tcf3*‐conditional and *Tcf12*‐conditional knockout groups in contemporaneous differentially expressed transcripts (Figure S2E). This indicated that the observable phenotypes are unique to TCF12. Compared to that in the control, 1094 transcripts were decreased in *Tcf12* ^*♀−/♂+*^ 2‐cell embryos (Figure 3A, Table S6). We then performed Gene Ontology (GO)‐based functional classification of the differentially expressed transcripts at the growing GV and 2‐cell stages. The results showed that the transcripts that were significantly dysregulated at the 2‐cell stage after *Tcf12* deletion were related to transcription, chromosome segregation, cell cycle, and blastocyst formation (Figure 3B). The transcripts that were significantly upregulated at the growing GV stage after *Tcf12* deletion were related to the negative regulation of transcription and growth, while the downregulated transcripts were related to transcription, exocytosis, and protein homotetramerisation (Figure 3B).

Fully grown GV oocytes isolated from PMSG‐treated WT and *Tcf12* ^*fl/fl*^;*Gdf9‐Cre* females were used for LC–MS/MS analysis to explore the direct triggers of polyspermy and early embryonic arrest induced by *Tcf12* (Table S7). All groups were sampled in duplicate and showed a high correlation (Figure S2C). Over 1500 proteins were detected in each group using quantitative mass spectrometry (Figure 3C). Volcano plot of mass spectrometry results indicated that 10 were significantly downregulated and 18 were significantly upregulated in all proteins examined in *Tcf12* ^*fl/fl*^;*Gdf9‐Cre* fully grown GV oocytes (Figure 3D, Table S8). Clustering analysis revealed that many differentially expressed proteins were related to fertilisation and cell‐cycle control (Figure 3E). We compared the RNA‐seq findings to the proteomic data and validated the deregulation of ASTL and ARPP19 at both the mRNA and protein levels (Figure 3F,G).

### 3.4 *Tcf12*‐Maternal Deletion Impairs the Expression and Cortical Localization of ASTL and Cortical Granules, Leading to Polyspermy

Next, we performed a functional validation of these target genes. During meiotic maturation, cortical granules migrate toward the cortex and form a “C‐shape” area localization at the MII stage and exocytose contents into the perivitelline space as soon as activation by sperm entry [38, 39]. Immature oocytes with insufficient or mis‐localised cortical granules cannot block polyspermy [40]. Ovastacin, encoded by *Astl*, is a cortical granule protease that accounts for the post‐fertilisation slit of the zona pellucida 2 (ZP2) to prevent excessive sperm‐binding in 2‐cell embryos [41]. Furthermore, deletion of *Astl* or mutation of the ZP2 cleavage site prevents ZP2 from post‐fertilisation cleavage and causes persistent sperm‐binding in 2‐cell embryos [42].

**FIGURE 4:**
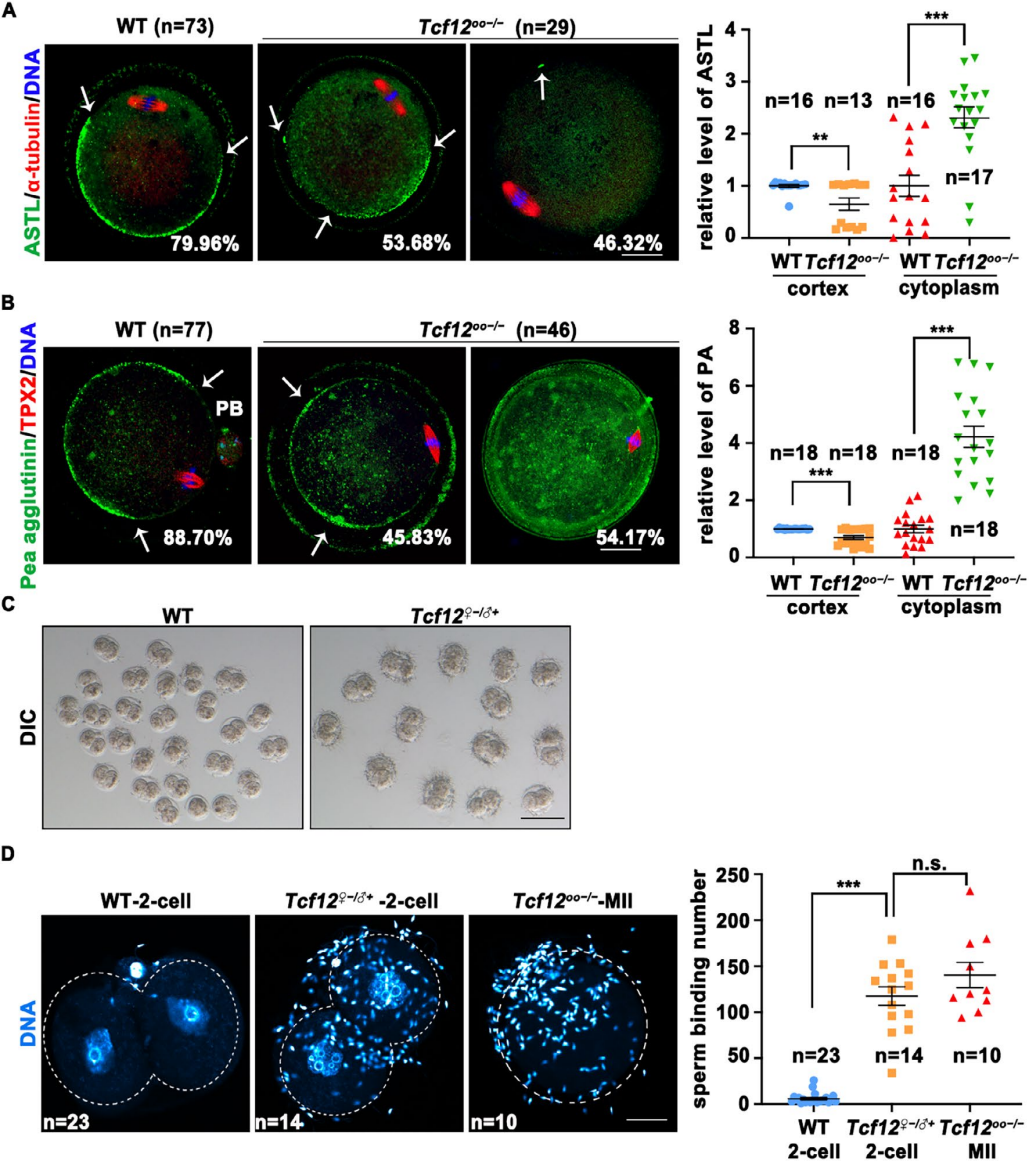
Maternal *Tcf12*‐deletion impairs the expression and cortical localization of ASTL and leads to polyspermy. (A, B) Immunofluorescence staining and statistical analysis of ASTL and pea agglutinin labelled cortical granules in WT and *Tcf12* ^*fl/fl*^;*Gdf9‐Cre* MII oocytes. Total numbers of analysed oocytes are indicated (*n*). The percentages of representative oocytes are shown in the corners. The borders of the fluorescent signal are indicated by white arrows. Scale bar, 50 μm. (C) Representative images of the MII oocytes and 2‐cell embryos which were derived from WT and *Tcf12* ^*fl/fl*^;*Gdf9‐Cre* female mice and incubated (0.5 h) with capacitated sperms. DIC, Differential interference contrast. Scale bar, 100 μm. (D) Immunofluorescence staining and statistical analysis showing the sperm‐binding number of the MII oocytes and 2‐cell embryos in (C). Dashed lines, cell outlines. Total numbers of analysed samples are indicated (*n*). Scale bar, 50 μm. Error bars, SEM; n.s., non‐significant; **p < 0.01; ***p < 0.001, computed using two‐tailed Student's *t*‐tests.

We examined the expression and cortical localisation of ASTL and cortical granules using immunofluorescence staining to test whether the polyspermy abnormality caused by a lack of *Tcf12* was associated with cortical granule exocytosis. Similar results were obtained for both ASTL‐ and pea agglutinin‐labelled cortical granules; the intensity of the cortical signals was notably reduced and became inhomogeneous in nearly half of *Tcf12* ^*fl/fl*^;*Gdf9‐Cre* MII oocytes. In the other half of *Tcf12* ^*fl/fl*^;*Gdf9‐Cre* oocytes, the intensity of the cortical signals was almost completely lost, whereas that of the intracytoplasmic signals increased (Figure 4A,B). Capacitated sperm could not bind to the zona pellucida of WT 2‐cell embryos (negative controls) in a 30‐min *de novo* sperm‐binding assay, but did bind to *Tcf12* ^*♀−/♂+*^2‐cell embryos and *Tcf12* ^*fl/fl*^;*Gdf9‐Cre* MII oocytes (positive controls) (Figure 4C,D). These results indicated that maternal *Tcf12* deletion impairs the expression and cortical localisation of ASTL and cortical granules, thus impeding hardening of the zona pellucida after fertilisation.

### 3.5 Ablation of TCF12 Disrupts the Cell Cycle of Zygotes by Promoting Premature Dephosphorylation of Histone H3, Possibly by Decreased PP2A‐B55 Inhibition

**FIGURE 5:**
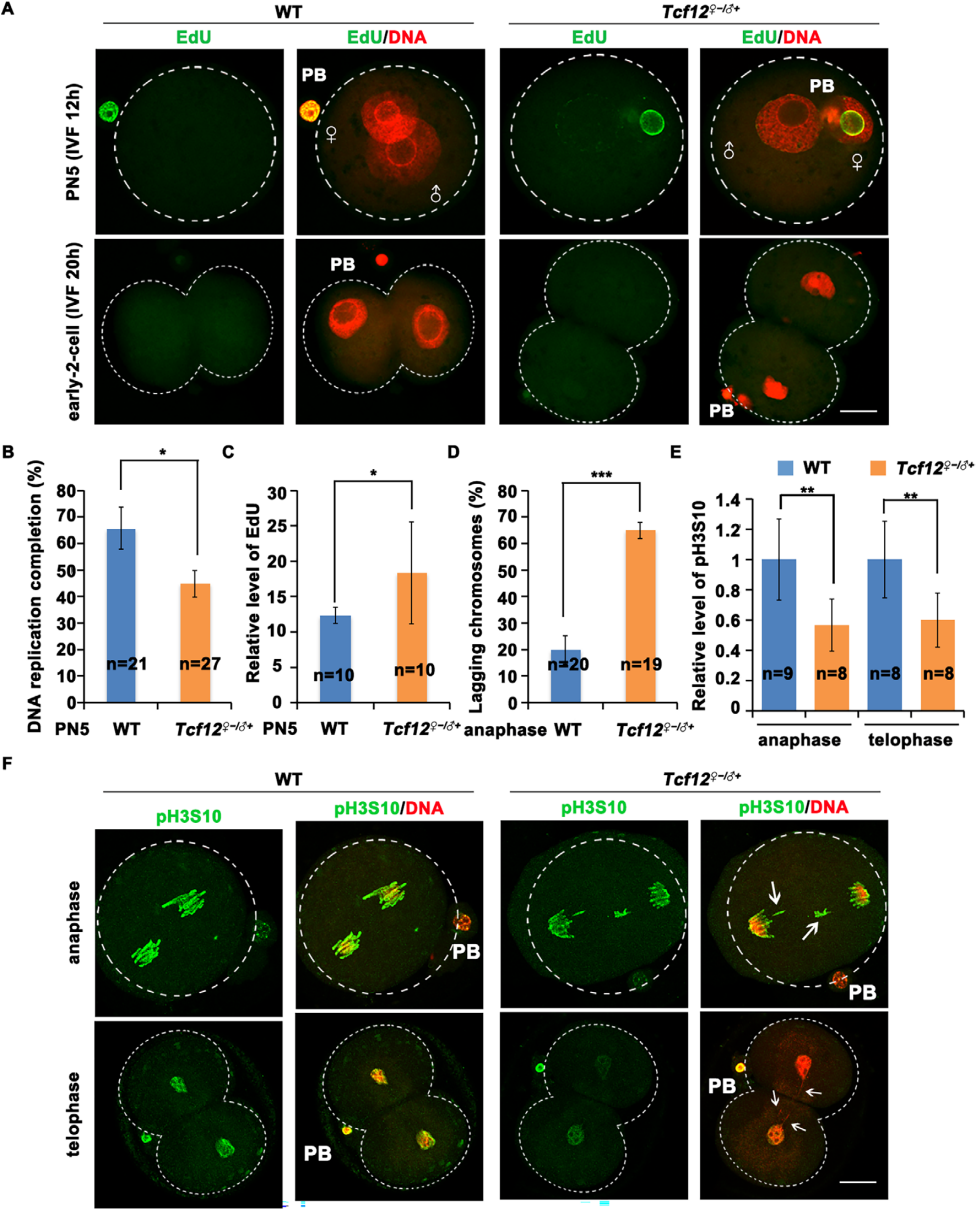
*Tcf12*‐maternal deletion impairs separation of sister chromatids, induced cell cycle delay of zygote. (A) Detection of DNA replication in WT and *Tcf12* ^*♀−/♂+*^ zygotes after IVF using 5‐ethynyl‐2′‐deoxy‐uridine (EdU) incorporation. PB: Polar body. The rates of representative oocytes are shown in the corners. Scale bar, 50 μm. (B) The proportion of PN5 that completes DNA replication in the WT and cKO groups based on EdU signals. Total numbers of analysed samples are indicated (*n*). (C) Relative fluorescence intensity statistics of EdU signal in PN5 of WT and cKO. Total numbers of analysed samples are indicated (*n*). (D) Proportion of fertilised eggs with late‐stage chromosome retention in the WT and cKO groups. Total numbers of analysed samples are indicated (*n*). (E) Relative fluorescence intensity statistics of the phosphorylation level of histone H3 on S10 (H3S10) signal of WT and cKO embryos. Total numbers of analysed samples are indicated (*n*). (F) Immunofluorescence staining showing the phosphorylation level of H3S10 of WT and *Tcf12* ^*♀−/♂+*^ zygotes after IVF. PB, Polar body. Premature detached sister chromatids are indicated using white arrows. Scale bar, 50 μm.

Because most *Tcf12* ^*♀−/♂+*^ embryos arrested at the 2‐cell stage, we analysed the first round of zygotic mitosis. We incubated zygotes with EdU, an analog of thymidine which can be incorporated into DNA strand during DNA replication, at different time points following in vitro fertilisation. Over half of the zygotes in the control groups completed replication 12 h after in vitro fertilisation and formed 2‐cell embryos with normal nuclear morphology 20 h after in vitro fertilisation (Figure 5A,B). In comparison, the *Tcf12* ^*♀−/♂+*^ zygotes developed slower than the control zygotes at indicated developmental stages (Figure 5C). We detected chromosome segregation using immunofluorescence to observe potential defects that occurred during the division phase. The chromosome condensation of *Tcf12* ^*♀−/♂+*^ zygotes appeared normal at mitosis entry, but it was partially lost during the anaphase to telophase (Figure 5D–F). The phosphorylation level of histone H3 on S10 (H3S10) was partially reduced in *Tcf12* ^*♀−/♂+*^ zygotes compared with that in the control, which accounted for the partial DNA decondensation (Figure 5E,F). This cell‐cycle related phenotype is similar to the reported phenotypes in *Arpp19* ^*−/−*^ mouse embryonic fibroblasts (MEFs). ARPP19 is a potent inhibitor of PP2A‐B55 during embryogenesis [43]. *Arpp19* knockout mice are embryonically lethal [43]. The absence of ARPP19 visibly prolonged the MEF cell cycle by premature dephosphorylation of cell cycle regulatory proteins during mitosis, probably due to reduced inhibition of PP2A‐B55 activity.

**FIGURE 6:**
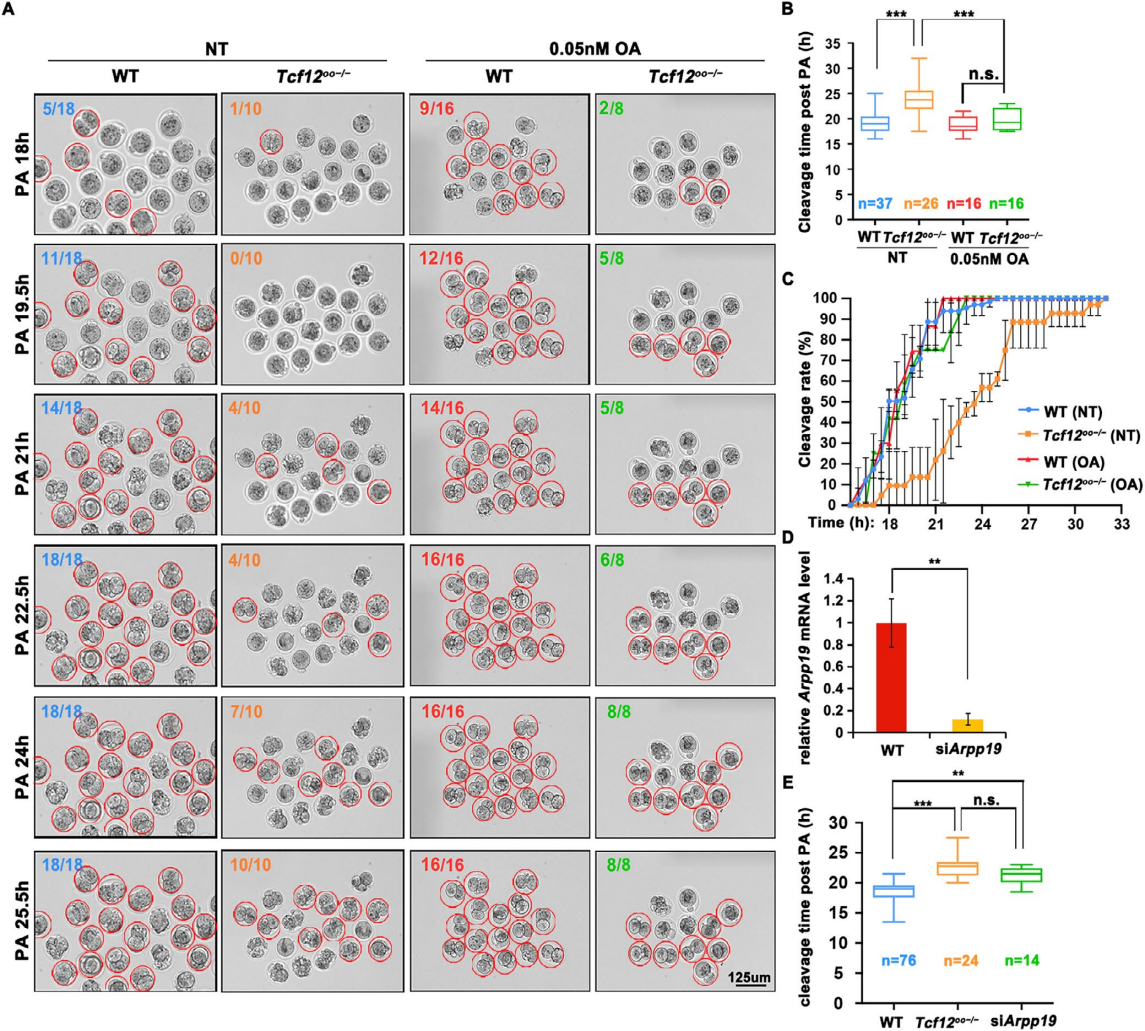
*Tcf12* ablation disrupts the cell cycle of zygotes by a decrease of PP2A‐B55 activity inhibition. (A) Representative images using time‐lapse imaging of WT and *Tcf12* ^*oo−/−*^parthenogenetic haploid embryos with different treatments. The numbers of embryos that reached the 2‐cell stage are shown in the corners. Parthenogenetic haploid embryos that complete the cleavage are indicated by red circles. Scale bar, 125 μm. (B, C) Cleavage time and cleavage rate of *siControl*, *Tcf12* ^*oo−/−*^ and *siArpp19* parthenogenetic haploid embryos. Total numbers of analysed samples are indicated (*n*). Error bars, SEM; n.s., non‐significant; **p < 0.01; ***p < 0.001, computed using two‐tailed Student's *t*‐tests. (D) Relative mRNA level of *Arpp19* in *siControl* and *siArpp19* fully grown GV oocytes measured using qRT‐PCR. *n* = 3 biological replicates. (E) Cleavage time of parthenogenetic haploid embryos in A at indicated collection sites. Total numbers of analysed samples are indicated (*n*).

We monitored the division of parthenogenetic haploid embryos with 0.05 nM okadaic acid (OA, a PP2A inhibitor) using time‐lapse microscopy to validate the hypothesis that the mitotic delay induced by *Tcf12* ablation arose from the insufficient inhibition of PP2A‐B55. Images were captured every 30 min (first acquisition at PA 12 h). Normally, cell division of WT parthenogenetic haploid embryos appeared at PA 19.5 h (23rd image acquisition), in contrast to PA 24 h (32nd image acquisition) of *Tcf12* ^*fl/fl*^;*Gdf9‐Cre* parthenogenetic embryos (Figure 6A–C). Adding OA rescued the abnormalities in cell cycle delay, suggesting that the observed knockout phenotypes were driven by alterations in PP2A‐B55 activity (Figure 6A–C). Moreover, validation of ARPP19 was also performed by injecting *Arpp19*‐targeting siRNAs into the cytoplasm of WT MII oocytes (Figure 6D). Similar to the *Tcf12* ^*fl/fl*^;*Gdf9‐Cre* parthenogenetic embryos, most *Arpp19* knockdown parthenogenetic embryos displayed a significant reduction in mitotic rate compared to the control (Figure 6E).

### 3.6 Maternal Depletion of TCF12 Disrupts ZGA in Preimplantation Embryos

**FIGURE 7:**
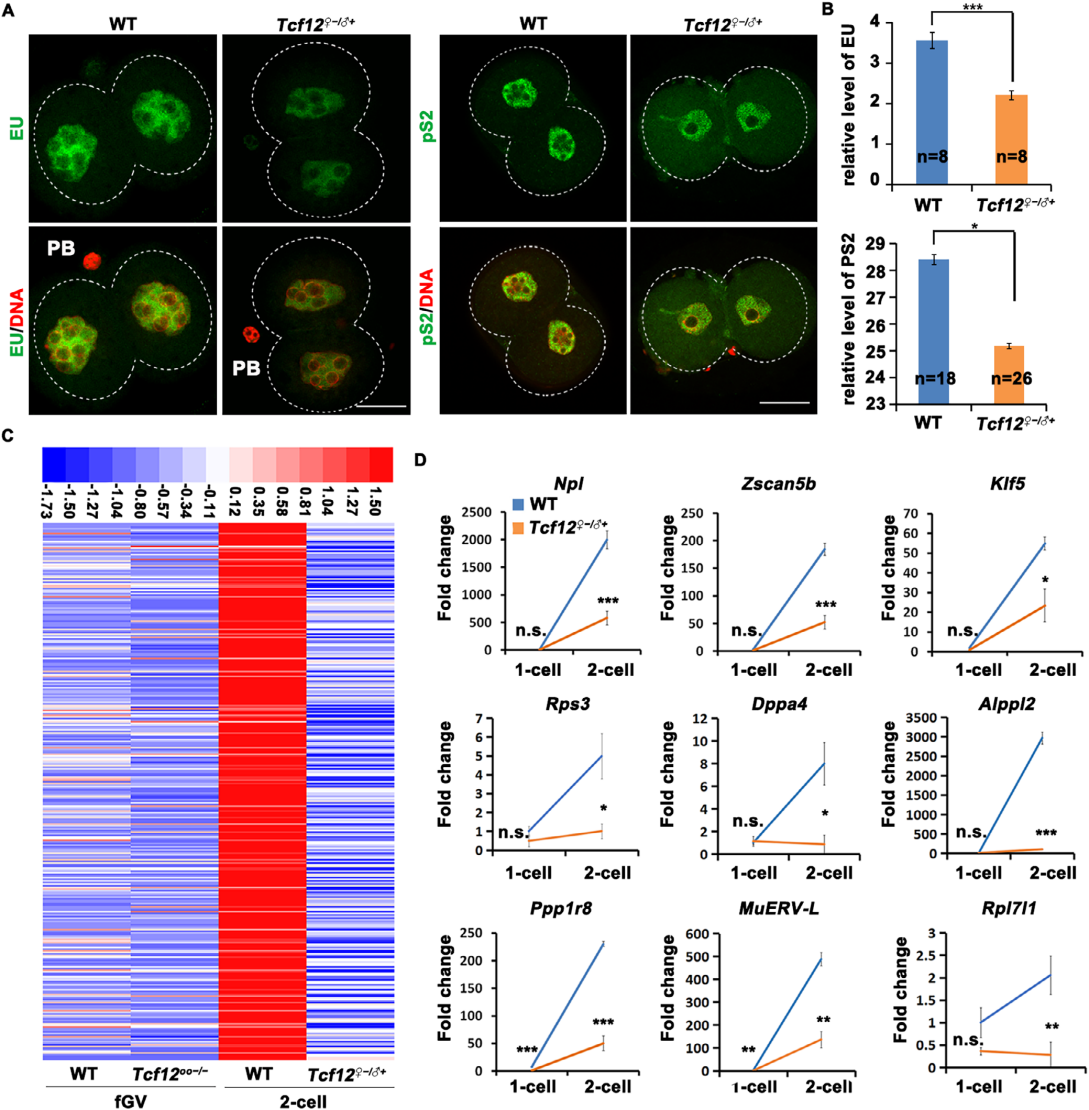
Maternal TCF12 Ensures the normal ZGA. (A and B) Immunofluorescence staining and statistical analysis of 5‐ethynyl uridine (EU) incorporation and phosphorylated RNA Polymerase II CTD (pS2) in WT and *Tcf12* ^*♀−/♂+*^ 2‐cell embryos. PB: Polar body. Total numbers of analysed samples are indicated (*n*). Scale bar, 50 μm. (C) Heatmap analysis showing the genes that are transcriptionally activated during ZGA in WT 2‐cell embryos but failed to be activated in *Tcf12* ^*♀−/♂+*^ embryos. (D) Relative mRNA levels of indicated ZGA marker genes at the zygote and 2‐cell stages in WT and *Tcf12* ^*♀−/♂+*^ embryos. *n* = 3 biological replicates. Error bars, SEM; n.s., non‐significant; *p < 0.05; **p < 0.01; ***p < 0.001, computed using two‐tailed Student's *t*‐tests.

As previously mentioned, 1094 transcripts were downregulated in *Tcf12* ^*♀−/♂+*^ 2‐cell embryos, most of which were related to transcription (Figure 3A,B). Accordingly, we proposed that the depletion of *Tcf12* causes a transient cell cycle delay followed by ZGA arrest. We performed EU staining and immunofluorescence of phosphorylated RNA Polymerase II CTD (pS2) in WT and *Tcf12* ^*♀−/♂+*^ 2‐cell embryos to detect newly synthesised RNAs during ZGA. The global transcription level of *Tcf12* ^*♀−/♂+*^ 2‐cell embryos was significantly lower than that in WT 2‐cell embryos (Figure 7A,B). More than half of the 1094 downregulated transcripts coincided with two previously obtained ZGA databases (Figure S2D) [44, 45]. Heatmap analysis of the expression of coincident genes confirmed the transcriptional inactivation of 572 downregulated transcripts in *Tcf12* ^*♀−/♂+*^ 2‐cell embryos (Figure 7C). This ZGA failure, caused by the lack of *Tcf12*, was further validated using qRT‐PCR of several representative ZGA genes (Figure 7D). These findings revealed that partial DNA decondensation‐induced cell cycle delay would cause impaired ZGA in *Tcf12* ^*♀−/♂+*^ 2‐cell embryos.

### 3.7 Deletion of *Tcf12* Impairs the Proper Expression of Differentiation‐Associated Genes Essential for Cell Fate Determination in Preimplantation Embryos

**FIGURE 8:**
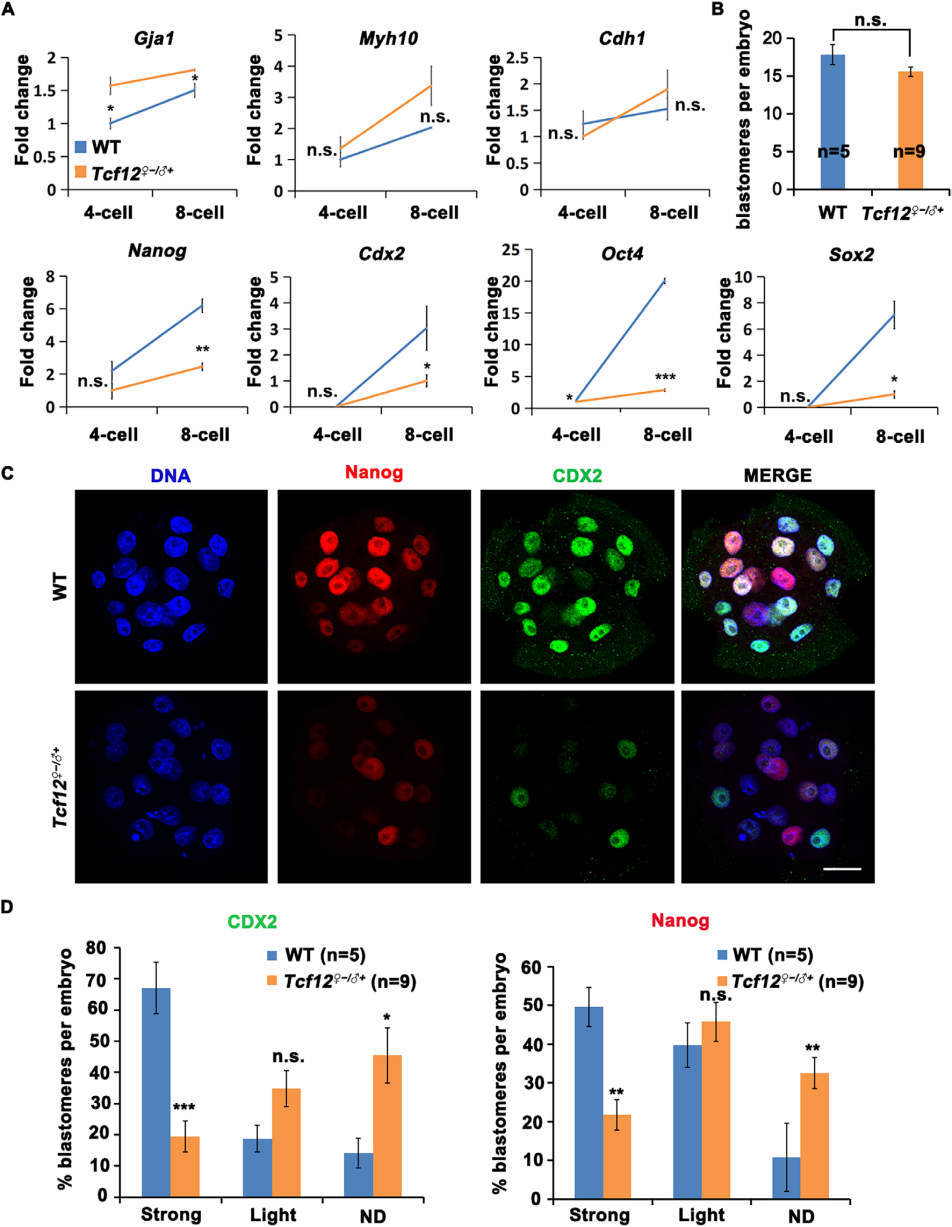
Maternal deletion of *Tcf12* impairs the proper expression of differentiation‐associated genes in preimplantation embryos. (A) Relative mRNA level of genes essential for cell fate commitment during the 4‐ to 8‐cell stages in WT and *Tcf12* ^*♀−/♂+*^ embryos. *n* = 3 biological replicates. (B) The number of blastomeres per embryo in WT and *Tcf12* ^*♀−/♂+*^ morula embryos. Total numbers of analysed samples are indicated (*n*). (C) Immunofluorescence staining showing the expression of NANOG and CDX2 in WT and *Tcf12* ^*♀−/♂+*^ morula embryos. Scale bar, 50 μm. (D) The rates of blastomeres with different signal intensities per embryo in WT and *Tcf12* ^*♀−/♂+*^ morula embryos. Total numbers of analysed samples are indicated (*n*). Error bars, SEM; n.s., non‐significant; *p < 0.05; **p < 0.01; ***p < 0.001, computed using two‐tailed Student's *t*‐tests.

The expression of genes essential for first‐cell lineage differentiation was activated from the 4‐cell to 8‐cell stage. Considering that few embryos could still develop into blastocysts, we determined the expression levels of genes essential for cell fate determination to predict embryo quality. Ablation of *Tcf12* did not alter the expression levels of *Gja1*, *Myh10*, and *Cdh1*, which were associated with embryonic compaction (Figure 8A). In contrast, the deletion of *Tcf12* caused aberrant transcriptional activation of differentiation‐associated genes (*Nanog*, *Cdx2*, *Oct4*, and *Sox2*) at the 8‐cell stage (Figure 8A). We then counted the numbers of blastomeres in in vitro cultured embryos for 96 h, and there was no difference between WT and *Tcf12* ^*♀−/♂+*^ embryos (Figure 8B). Immunofluorescence and statistical analysis showed that the concentration of NANOG and CDX2 decreased substantially in *Tcf12* ^*♀−/♂+*^ embryos at the morula stage (Figure 8C,D). Especially, the number of highly CDX2 or NANOG expressing blastomeres was reduced in *Tcf12* ^*♀−/♂+*^ morula embryos (Figure 8D). These results suggested that *Tcf12* ^*♀−/♂+*^ embryos lose the differentiation potential during development. As the defects in cortical granule localization and fertilisation were clearly detected in *Tcf12* KO oocytes, it is highly possible that the observed phenotype in fertilised embryos (altered ZGA and reduced expression of lineage‐specific markers) is merely secondary to these defects in oocytes. This can be explained by the absence of TCF12 in post‐fertilisation embryos including 2‐cell embryos (Figure 1D,E).

## 4 Discussion

**FIGURE 9:**
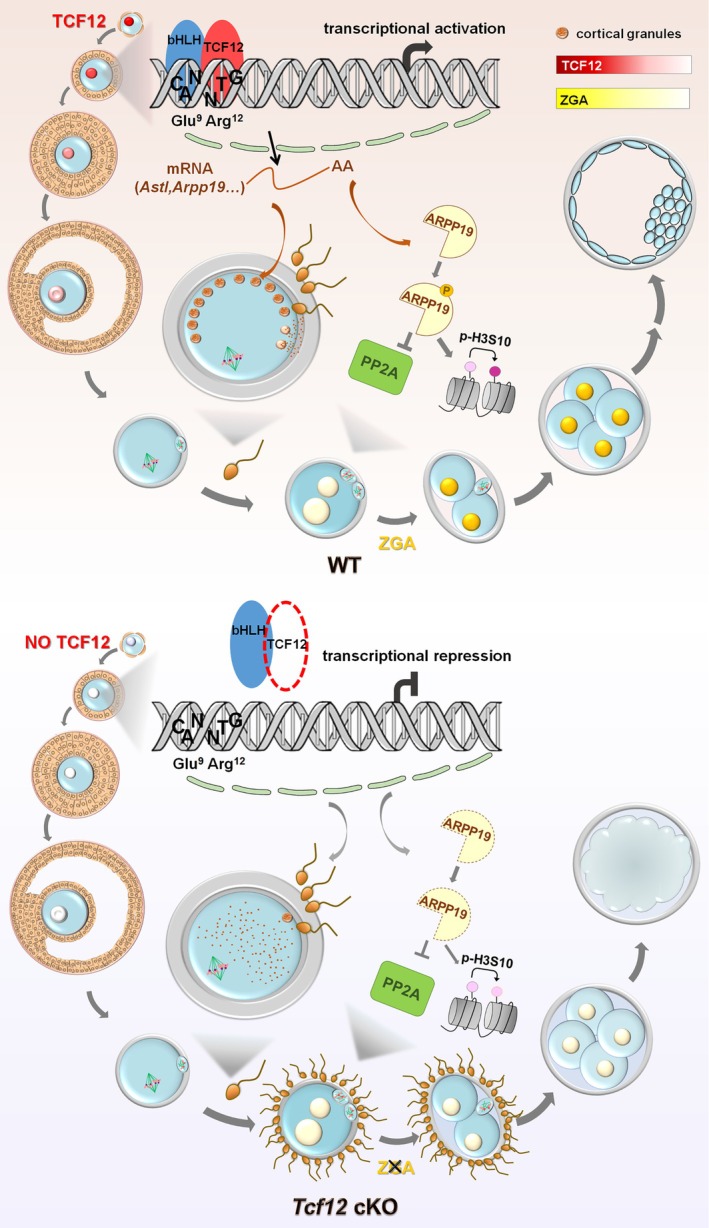
In vivo function of TCF12 in oocyte development and maternal‐zygotic transition (MZT). As a transcription factor abundantly expressed in the nucleus of oocytes during the growing phase, TCF12 moderates the transcription of selective target genes whose protein products are not essential during oocyte growth and meiotic maturation but are required for fertilisation and preimplantation embryo development. Maternal TCF12 ensures the competence of fertilisation by controlling the expression of *Astl* and the proper location of cortical granules. Furthermore, maternal TCF12 maintains the phosphatase activity of PP2A by regulating the expression of *Arpp19*. Oocyte‐specific deletion of *Tcf12* leads to fertilisation defects as well as ZGA failure at the 2‐cell stage. Consequently, maternal TCF12 is essential for successful maternal‐to‐zygotic transition and female fertility.

In our study, we demonstrated that the E protein family members, *Tcf12* and *Tcf3*, are abundantly expressed in the nuclei of premature oocytes and play crucial roles in the accumulation of maternal mRNA. Previous studies have shown that heterodimers formed by TCF12 and TCF3 regulate follicular growth‐related gene transcription, indicating that TCF3 and TCF12 have compensatory roles and imply that they jointly play crucial roles in the development of oocytes in primordial follicles. Notably, homodimer formation allows TCF12 and TCF3 to perform unique functions within the female reproductive system. TCF3 is a maternal transcription factor that contributes to meiotic maturation, maternal mRNA degradation, and epigenetic reprogramming of oocytes. We also analysed the phenotypes of Tcf3 knockout mice. These mice also displayed female infertility. Different from Tcf12 knockout mice, however, oocyte meiotic maturation is impaired after TCF3 deletion. During oocyte maturation, TCF3 depletion affects maternal mRNA clearance and H3K4me3 accumulation, causing defects in spindle assembly and equatorial chromosome alignment (L.J., unpublished data). Our study showed that *Tcf12* is a maternal transcription factor specifically expressed in growing and fully grown oocytes. Despite its function during oocyte growth, *Tcf12* is not important for folliculogenesis and meiotic maturation but is essential for fertilisation and early embryonic development. Unlike the maternal transcription factors HSF1 and USF1, the role of TCF12 in embryos is indirect. TCF12 is required to regulate the expression of maternal *Astl* and *Arpp19* during the growth phase, which function in the fertilisation and cell cycle exit processes, respectively (Figure 9).

Capacitated sperms recognise and penetrate the zona pellucida of MII oocytes in the fallopian tube. The mechanisms that block polyspermy, which leads to embryonic lethality, are equally significant [46, 47, 48]. The polyspermy block relies on cortical granules and membrane‐bound vesicles, which are derived from the Golgi apparatus and exocytose proteases into the perivitelline space upon activation [49, 50]. ASTL is a cortical granule protease that accounts for the hardening of the zona pellucida post‐fertilisation. *Tcf12*‐maternal deletion MII oocytes were deficient in their ability to block polyspermy, with disordered disruption of the cortical granules and ASTL.

Even after fertilisation, large numbers of *Tcf12* conditional knockout embryos arrested at the 2‐cell stage as a result of ZGA failure, possibly due to the first mitotic cell cycle extension. Mitotic cell cycle entry and exit are regulated by a balance between the activity of CDK1 and phosphatase PP2A‐B55 [51, 52, 53]. *Tcf12* ablation disrupted the cell cycle of zygotes by decreasing the expression of *Arpp19* and inhibiting PP2A‐B55 activity These maternal defects, induced by the deletion of *Tcf12*, reduced embryo viability.

Thus, we systematically studied transcriptome regulation by TCF12 during oogenesis and early embryonic development. This result suggested that maternal transcription factors do not necessarily affect oocyte meiotic maturation and that zygotic transcription factors do not necessarily affect ZGA. In this study, we clarified the role of maternal effects on early embryonic development, providing a new theoretical basis for clinical infertility problems and the advancement of assisted reproductive technology.

## Author Contributions

H.‐Y.F., J.L. and H.‐B.W. conceived the project. H.‐Y.F., J.L. H.‐B.W. and L.‐R.C. designed and analyzed experiments. L.‐R.C., C.Z., Z.‐Q.D., Y.‐X.Q. and Z.Z. performed experiments. H.‐Y.F., J.L. and H.‐B.W. provided key reagents and materials. L.‐R.C. and H.‐Y.F. wrote the paper.

## Conflicts of Interest

The authors declare no conflicts of interest.

## Supporting information


**Data S1:** Supporting Information.

**Table S5:** FPKMs of RNA‐seq results (in a separate xlsx file).

**Table S6:** FPKMs of transcripts decreased or increased for more than 2 folds in *Tcf12* ^−/−^ oocyte or *Tcf12* ^*♀−/♂+*^ embryo samples (in a separate xlsx file).

**Table S7:** Detected proteins of LC–MS/MS analysis (in a separate xlsx file).

**Table S8:** Detected proteins decreased or increased for more than 2 folds in *Tcf12* ^−/−^ oocyte samples (in a separate xlsx file).

## Data Availability

The RNA‐seq data were deposited in the NCBI Gene Expression Omnibus database under the accession code GSE211286. GEO accession number: GSE211286 at https://www.ncbi.nlm.nih.gov/geo/query/acc.cgi?acc=GSE211286. Please input the token cjypyuyujbwnjeh in the designated field. Mass spectrometry proteomic data are available from the ProteomeXchange Consortium via the iProX repository under the dataset identifier PXD046728 (http://proteomecentral.proteomexchange.org).
