# Supplementary material for: Transcription Factor TCF12‐Mediated Maternal Gene Expressions in Mouse Oocyte Are Prerequisites of Successful Fertilisation and Zygotic Genome Activation
Source: Cell Prolif. 2025 Aug 3;59(3):e70110. doi: 10.1111/cpr.70110 (PMC12961549; doi:10.1111/cpr.70110)
Supplement: Supplementary file 1 — Data S1: Supporting Information. [file CPR-59-e70110-s005.docx]

**Supplemental Information**

**Supplemental Figures**

**
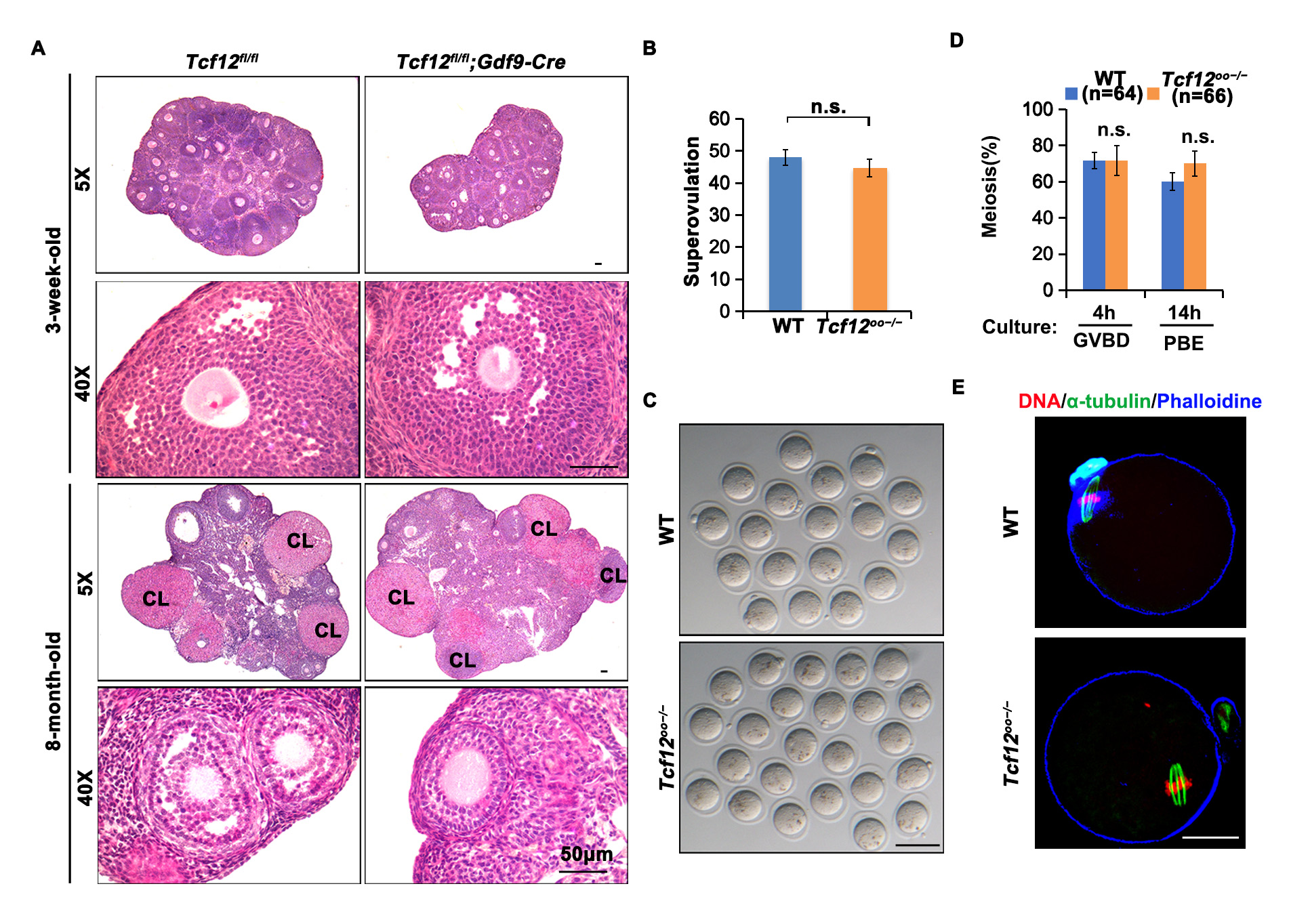
**

**Figure S1: *Tcf12*-deletion in oocytes does not affect folliculogenesis and meiotic maturation. A:** hematoxylin-eosin staining (H&E) results showing the ovarian histology of 3-week and 8-month-old female mice with indicated genotypes. Scale bar, 50 μm. **B:** Numbers of eggs being ovulated by 4-week old WT and *Tcf12^fl/fl^;Gdf9-Cre* female mice after superovulation. **C:** Representative images of ovulated eggs derived from the oviducts of WT and *Tcf12^fl/fl^;Gdf9-Cre* female mice after superovulation. Scale bar, 100 μm. n = 5 biological replicates each group. **D:** Percentage of the fully grown oocytes that cultured *in vitro* and underwent germinal vesicle breakdown (GVBD) and polar body-1 (PB1) emission at indicated time points. Fully grown GV oocytes were collected from PMSG-treated (44 h) female mice of the indicated genotypes. Total numbers of analyzed oocytes are indicated (n). Error bars, SEM; n.s., non-significant, computed by two-tailed Student’s *t*-tests. **E:** IF results showing spindle assembly of oocytes cultured *in vitro* for 14 hours. Scale bar, 50 μm.


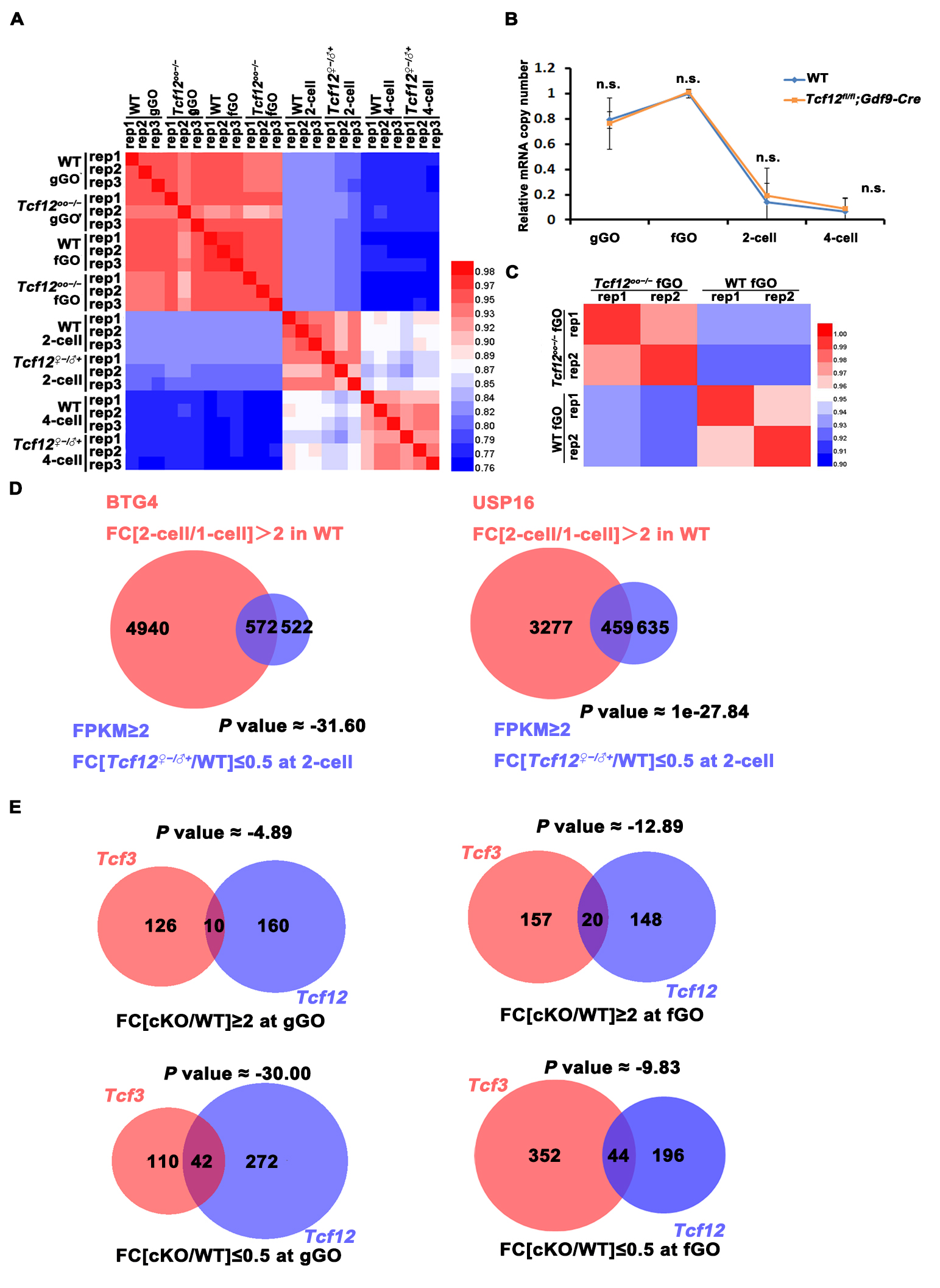


**Figure S2: Transcriptome analyses in oocytes and early embryos of *Tcf12^fl/fl^;Gdf9-Cre* mice. A:** Heatmap of correlation analysis in transcriptome between WT and *Tcf12^fl/fl^;Gdf9-Cre* oocytes and embryos at indicated stages. **B:** Relative total mRNA copy number of WT and *Tcf12^fl/fl^;Gdf9-Cre* oocytes and embryos at indicated stages. **C:** Heatmap of correlation analysis in proteome between WT and *Tcf12^fl/fl^;Gdf9-Cre* fully grown GV oocytes. **D:** Venn diagram showing the overlap between the transcripts activated during 1-cell-to-2-cell transition in WT and the transcripts that are decreased in *Tcf12^♀–/♂+^* 2-cell embryos compared to control. **E:** Venn diagram showing the overlap of differentially expressed transcripts between *Tcf3*-conditional knockout group and *Tcf12*-conditional knockout group at growing and fully grown GV stages.

**Supplemental Tables**

**Table S1. Antibody information.**

| Protein name | Manufacture  (catalogue number) | Applications  (working dilution) |
| --- | --- | --- |
| DDB1 | Epitomics (3821-1) | WB (1:5000) |
| FITC-α-tubulin | Sigma (F2168) | IF (1:200) |
| TCF12 | Proteintech (14419-1-AP) | IHC (1:200); IF(1:200) |
| ASTL | Invitrogen（PA5-53343） | IF(1:200); WB(1:1000) |
| ARPP19 | Proteintech (11678-1-AP) | WB(1:1000) |
| Pea agglutinin | Sigma (L0770) | IF(1:200) |
| pH3S10 | Cell Signaling (9701S) | IF(1:200) |
| Phosphorylated RNA Pol II (Ser2P) | Abcam (ab5095) | IF(1:400) |
| Nanog | Cell Signaling (8822S) | IF (1:200) |
| CDX2 | BioGenex (AM392-5M) | IF (Ready to use) |
| pH2A.X | Cell Signaling(9718S) | IF (1:400) |
| Phalloidine | Invitrogen (R415) | IF (1:400) |
| ZP2 | Sigma (AV45470) | WB(1:1000) |

**Table S2. Primer sequences.**

| Target Gene | Application | Sequences (5′-3′) |
| --- | --- | --- |
| *Tcf12* | Genotyping | 5’ - CCGTGGCAGTCATCCTTAGT-3’ |
|  |  | 5’ - TAACTGCCCAGCACAGAAGA-3’ |
| *Gdf9-Cre* | Genotyping | 5’- TCTGATGAAGTCAGGAAGAACC-3’ |
|  |  | 5’- GAGATGTCCTTCACTCTGATTC-3’ |
| *Tcf12* | qRT-PCR | 5’- CTGACCTTTGGAGTTCATCGAATGG-3’ |
|  |  | 5’-CATTGATGGGAGGAGTATGTGAGGC-3’ |
| *Tcf3* | qRT-PCR | 5’-GCCCACTTCAGTGACTCCCACAG-3’ |
|  |  | 5’-CCATCTGCAGCTCTCCGACGAG-3’ |
| *Flag*-*Gfp*-*Tcf12_RT_* | qRT-PCR | 5’-GCTCTAGACTGACCTTTGGAGTTCATCGAATG-3’ |
|  |  | 5’-ACGCGTCGACCATTGATGGGAGGAGTATGTGAGGC-3’ |
| *Flag*-*Gfp*-*Tcf3_RT_* | qRT-PCR | 5’-GCTCTAGAGCCCACTTCAGTGACTCCCACAG-3’ |
|  |  | 5’-ACGCGTCGACCCATCTGCAGCTCTCCGACGAG-3’ |
| *Flag*-*Gfp-RT* | qRT-PCR | 5’-GGGTGGTGCCCATCCTGGTC-3’ |
|  |  | 5’-CCTGGACGTAGCCTTCGGGC-3’ |
| *Arpp19* | qRT-PCR | 5’-CAAGGTATCCTCACTTGGGACA-3’ |
|  |  | 5’-GGTCCTGTGGAGTGGGAATG-3’ |
| *Astl* | qRT-PCR | 5’- CTGAGGGAAGCCCGGTATTTC-3’ |
|  |  | 5’- GCCGGATAATGTCCCCTTCTA-3’ |
| *Zp2* | qRT-PCR | 5’-GCAGCTGGAGCTCTTGTTCT-3’ |
|  |  | 5’-TCCATTGTCCAAAGTCCACA-3’ |
| *Npl* | qRT-PCR | 5’-AAGAAACTCCGGGGTCTTGTT-3’ |
|  |  | 5’-CCTGTGGTGCCATTCACAAAAAT-3’ |
| *Zscan5b* | qRT-PCR | 5’-ATGGGCAATACAGAAGATGGGC-3’ |
|  |  | 5’-GGTCAAACCGGGACTTGTAAA-3’ |
| *Klf5* | qRT-PCR | 5’-ACGTACACCATGCCAAGTCA-3’ |
|  |  | 5’-GTGGGAGAGTTGGCGAATTA-3’ |
| *Ppp1r8* | qRT-PCR | 5’-AACCCCGATCTGTGTGACTTC-3’ |
|  |  | 5’-CCAGCCGAATGTGACCCAA-3’ |
| *MuERV-L* | qRT-PCR | 5’-ATCGAAAGGCTCCAGACACAA-3’ |
|  |  | 5’-TTCAGCCAACCTTACAATGAGAG-3’ |
| *Rps3* | qRT-PCR | 5’-ATGGCGGTGCAGATTTCCAA-3’ |
|  |  | 5’-GTAACTCGGACTTCAACTCCAG-3’ |
| *Dppa4* | qRT-PCR | 5’-AGTCAACCTAGCACGGCTC-3’ |
|  |  | 5’-TCCTGGCGTCTCAGTGTCT-3’ |
| *Alppl2* | qRT-PCR | 5’-GAGCGTCATCCCAGTGGAG-3’ |
|  |  | 5’-TAGCGGTTACTGTAGACACCC-3’ |
| *Rpl6* | qRT-PCR | 5’-AAGCCCAAGAAGGCGAAGC-3’ |
|  |  | 5’-GCAGCCGAGTATTTCCTTTTGTA-3’ |
| *Rpl7l1* | qRT-PCR | 5’-CGACTAGAATCTTTCGTGCATGA-3’ |
|  |  | 5’-GGGATGTTTATCAGGCACCTC-3’ |
| *Nid2* | qRT-PCR | 5’-CACCGAGGACAGTTTCCATT-3’ |
|  |  | 5’-CCAGTTACCAGGTGCTGGAT-3’ |
| *Rpl38* | qRT-PCR | 5’-TGCCAAGTCTGTCAAGATCAAG-3’ |
|  |  | 5’-TGTCTGTGATAACCAGGGTGTA-3’ |
| *Rpl39* | qRT-PCR | 5’-ATGTCTTCTCACAAGACTTTCCGAATCAA-3’ |
|  |  | 5’-TTACAGACCCAGCTTCGTTCTCCTCC-3’ |
| *Gm7102* | qRT-PCR | 5’-AGGCTCCTACTCCAGGTCC-3’ |
|  |  | 5’-ACGCCCTGATCCAAATGGTG-3’ |
| *Ctsl* | qRT-PCR | 5’-ATCAAACCTTTAGTGCAGAGTGG-3’ |
|  |  | 5’-CTGTATTCCCCGTTGTGTAGC-3’ |
| *Rpl10l* | qRT-PCR | 5’-ACCCAAAGTCCCGTTTCTGC-3’ |
|  |  | 5’-CTCGTCCGACACCATGTGG-3’ |
| *Nanog* | qRT-PCR | 5’-TTCTTGCTTACAAGGGTCTGC-3’ |
|  |  | 5’-AGAGGAAGGGCGAGGAGA-3’ |
| *Cdx2* | qRT-PCR | 5’-CAAGGACGTGAGCATGTATCC-3’ |
|  |  | 5’-GTAACCACCGTAGTCCGGGTA-3’ |
| *Oct4* | qRT-PCR | 5’-ATGGGGAAAGAAGCTCAGTG-3’ |
|  |  | 5’-CAAAATGATGAGTGACAGACAGG-3’ |
| *Sox2* | qRT-PCR | 5’-GCGGAGTGGAAACTTTTGTCC-3’ |
|  |  | 5’-CGGGAAGCGTGTACTTATCCTT-3’ |
| *Gja1* | qRT-PCR | 5’-ACAGCGGTTGAGTCAGCTTG-3’ |
|  |  | 5’-GAGAGATGGGGAAGGACTTGT-3’ |
| *Myh10* | qRT-PCR | 5’-GGAATCCTTTGGAAATGCGAAGA-3’ |
|  |  | 5’-GCCCCAACAATATAGCCAGTTAC-3’ |
| *Cdh1* | qRT-PCR | 5’-CAGGTCTCCTCATGGCTTTGC-3’ |
|  |  | 5’-CTTCCGAAAAGAAGGCTGTCC-3’ |
| *Gapdh* | qRT-PCR | 5’-ACACTGAGGACCAGGTTGTCTC-3’ |
|  |  | 5’-TACTCCTTGGAGGCCATGTAG-3’ |

**Table S3. Spearman correlation coefficients among WT and *Tcf12^oo^*^−/−^ oocytes or *Tcf12^♀−/♂+^* embryos*.***

| Stage | Sample | | gGO | | | | | | fGO | | | | | | 2-cell | | | | | | 4-cell | | | | | |
| --- | --- | --- | --- | --- | --- | --- | --- | --- | --- | --- | --- | --- | --- | --- | --- | --- | --- | --- | --- | --- | --- | --- | --- | --- | --- | --- |
|  |  |  | WT | | | *Tcf12^oo−/−^* | | | WT | | | *Tcf12^oo−/−^* | | | WT | | | *Tcf12^♀−/♂+^* | | | WT | | | *Tcf12^♀−/♂+^* | | |
|  |  |  | 1 | 2 | 3 | 1 | 2 | 3 | 1 | 2 | 3 | 1 | 2 | 3 | 1 | 2 | 3 | 1 | 2 | 3 | 1 | 2 | 3 | 1 | 2 | 3 |
| gGO | WT | 1 | 1.00 | 1.00 | 0.99 | 0.98 | 0.60 | 0.97 | 0.95 | 0.95 | 0.96 | 0.93 | 0.93 | 0.93 | 0.43 | 0.39 | 0.40 | 0.45 | 0.28 | 0.44 | 0.38 | 0.38 | 0.39 | 0.37 | 0.39 | 0.40 |
|  |  | 2 | 1.00 | 1.00 | 0.99 | 0.98 | 0.65 | 0.96 | 0.94 | 0.94 | 0.96 | 0.92 | 0.92 | 0.92 | 0.48 | 0.42 | 0.42 | 0.50 | 0.34 | 0.47 | 0.42 | 0.43 | 0.43 | 0.40 | 0.44 | 0.44 |
|  |  | 3 | 0.99 | 0.99 | 1.00 | 0.98 | 0.58 | 0.98 | 0.95 | 0.96 | 0.96 | 0.93 | 0.94 | 0.94 | 0.43 | 0.40 | 0.41 | 0.44 | 0.27 | 0.44 | 0.38 | 0.37 | 0.38 | 0.37 | 0.38 | 0.39 |
|  | *Tcf12^oo−/−^* | 1 | 0.98 | 0.98 | 0.98 | 1.00 | 0.62 | 0.99 | 0.95 | 0.96 | 0.97 | 0.95 | 0.95 | 0.96 | 0.46 | 0.43 | 0.44 | 0.47 | 0.30 | 0.48 | 0.43 | 0.42 | 0.43 | 0.42 | 0.44 | 0.44 |
|  |  | 2 | 0.60 | 0.65 | 0.58 | 0.62 | 1.00 | 0.53 | 0.51 | 0.51 | 0.52 | 0.51 | 0.49 | 0.49 | 0.71 | 0.44 | 0.42 | 0.82 | 0.89 | 0.59 | 0.61 | 0.76 | 0.67 | 0.53 | 0.71 | 0.71 |
|  |  | 3 | 0.97 | 0.96 | 0.98 | 0.99 | 0.53 | 1.00 | 0.96 | 0.97 | 0.96 | 0.95 | 0.96 | 0.97 | 0.40 | 0.40 | 0.41 | 0.39 | 0.22 | 0.43 | 0.38 | 0.35 | 0.37 | 0.37 | 0.37 | 0.37 |
| fGO | WT | 1 | 0.95 | 0.94 | 0.95 | 0.95 | 0.51 | 0.96 | 1.00 | 0.99 | 0.99 | 0.99 | 0.99 | 0.99 | 0.42 | 0.44 | 0.45 | 0.41 | 0.20 | 0.45 | 0.34 | 0.33 | 0.34 | 0.34 | 0.34 | 0.35 |
|  |  | 2 | 0.95 | 0.94 | 0.96 | 0.96 | 0.51 | 0.97 | 0.99 | 1.00 | 0.99 | 0.99 | 0.99 | 0.99 | 0.41 | 0.43 | 0.44 | 0.39 | 0.20 | 0.45 | 0.34 | 0.33 | 0.34 | 0.34 | 0.34 | 0.35 |
|  |  | 3 | 0.96 | 0.96 | 0.96 | 0.97 | 0.52 | 0.96 | 0.99 | 0.99 | 1.00 | 0.98 | 0.98 | 0.98 | 0.42 | 0.43 | 0.44 | 0.41 | 0.20 | 0.45 | 0.35 | 0.34 | 0.35 | 0.35 | 0.35 | 0.36 |
|  | *Tcf12^oo−/−^* | 1 | 0.93 | 0.92 | 0.93 | 0.95 | 0.51 | 0.95 | 0.99 | 0.99 | 0.98 | 1.00 | 0.99 | 0.99 | 0.42 | 0.44 | 0.46 | 0.41 | 0.20 | 0.46 | 0.36 | 0.34 | 0.36 | 0.36 | 0.35 | 0.36 |
|  |  | 2 | 0.93 | 0.92 | 0.94 | 0.95 | 0.49 | 0.96 | 0.99 | 0.99 | 0.98 | 0.99 | 1.00 | 1.00 | 0.41 | 0.43 | 0.45 | 0.39 | 0.19 | 0.45 | 0.35 | 0.33 | 0.35 | 0.35 | 0.34 | 0.35 |
|  |  | 3 | 0.93 | 0.92 | 0.94 | 0.96 | 0.49 | 0.97 | 0.99 | 0.99 | 0.98 | 0.99 | 1.00 | 1.00 | 0.40 | 0.43 | 0.45 | 0.38 | 0.18 | 0.46 | 0.35 | 0.32 | 0.34 | 0.35 | 0.34 | 0.35 |
| 2-cell | WT | 1 | 0.43 | 0.48 | 0.43 | 0.46 | 0.71 | 0.40 | 0.42 | 0.41 | 0.42 | 0.42 | 0.41 | 0.40 | 1.00 | 0.90 | 0.88 | 0.94 | 0.76 | 0.93 | 0.61 | 0.72 | 0.66 | 0.52 | 0.68 | 0.71 |
|  |  | 2 | 0.39 | 0.42 | 0.40 | 0.43 | 0.44 | 0.40 | 0.44 | 0.43 | 0.43 | 0.44 | 0.43 | 0.43 | 0.90 | 1.00 | 0.99 | 0.73 | 0.44 | 0.95 | 0.48 | 0.53 | 0.51 | 0.43 | 0.51 | 0.55 |
|  |  | 3 | 0.40 | 0.42 | 0.41 | 0.44 | 0.42 | 0.41 | 0.45 | 0.44 | 0.44 | 0.46 | 0.45 | 0.45 | 0.88 | 0.99 | 1.00 | 0.71 | 0.41 | 0.95 | 0.50 | 0.52 | 0.51 | 0.45 | 0.51 | 0.55 |
|  | *Tcf12^♀−/♂+^* | 1 | 0.45 | 0.50 | 0.44 | 0.47 | 0.82 | 0.39 | 0.41 | 0.39 | 0.41 | 0.41 | 0.39 | 0.38 | 0.94 | 0.73 | 0.71 | 1.00 | 0.88 | 0.84 | 0.59 | 0.74 | 0.65 | 0.48 | 0.68 | 0.70 |
|  |  | 2 | 0.28 | 0.34 | 0.27 | 0.30 | 0.89 | 0.22 | 0.20 | 0.20 | 0.20 | 0.20 | 0.19 | 0.18 | 0.76 | 0.44 | 0.41 | 0.88 | 1.00 | 0.61 | 0.57 | 0.76 | 0.64 | 0.46 | 0.69 | 0.69 |
|  |  | 3 | 0.44 | 0.47 | 0.44 | 0.48 | 0.59 | 0.43 | 0.45 | 0.45 | 0.45 | 0.46 | 0.45 | 0.46 | 0.93 | 0.95 | 0.95 | 0.84 | 0.61 | 1.00 | 0.62 | 0.67 | 0.64 | 0.55 | 0.65 | 0.68 |
| 4-cell | WT | 1 | 0.38 | 0.42 | 0.38 | 0.43 | 0.61 | 0.38 | 0.34 | 0.34 | 0.35 | 0.36 | 0.35 | 0.35 | 0.61 | 0.48 | 0.50 | 0.59 | 0.57 | 0.62 | 1.00 | 0.93 | 0.96 | 0.97 | 0.95 | 0.95 |
|  |  | 2 | 0.38 | 0.43 | 0.37 | 0.42 | 0.76 | 0.35 | 0.33 | 0.33 | 0.34 | 0.34 | 0.33 | 0.32 | 0.72 | 0.53 | 0.52 | 0.74 | 0.76 | 0.67 | 0.93 | 1.00 | 0.98 | 0.89 | 0.99 | 0.99 |
|  |  | 3 | 0.39 | 0.43 | 0.38 | 0.43 | 0.67 | 0.37 | 0.34 | 0.34 | 0.35 | 0.36 | 0.35 | 0.34 | 0.66 | 0.51 | 0.51 | 0.65 | 0.64 | 0.64 | 0.96 | 0.98 | 1.00 | 0.94 | 0.99 | 0.99 |
|  | *Tcf12^♀−/♂+^* | 1 | 0.37 | 0.40 | 0.37 | 0.42 | 0.53 | 0.37 | 0.34 | 0.34 | 0.35 | 0.36 | 0.35 | 0.35 | 0.52 | 0.43 | 0.45 | 0.48 | 0.46 | 0.55 | 0.97 | 0.89 | 0.94 | 1.00 | 0.93 | 0.92 |
|  |  | 2 | 0.39 | 0.44 | 0.38 | 0.44 | 0.71 | 0.37 | 0.34 | 0.34 | 0.35 | 0.35 | 0.34 | 0.34 | 0.68 | 0.51 | 0.51 | 0.68 | 0.69 | 0.65 | 0.95 | 0.99 | 0.99 | 0.93 | 1.00 | 0.99 |
|  |  | 3 | 0.40 | 0.44 | 0.39 | 0.44 | 0.71 | 0.37 | 0.35 | 0.35 | 0.36 | 0.36 | 0.35 | 0.35 | 0.71 | 0.55 | 0.55 | 0.70 | 0.69 | 0.68 | 0.95 | 0.99 | 0.99 | 0.92 | 0.99 | 1.00 |

**Table S4. Quality control of WT and *Tcf12*^−/−^ oocytes or *Tcf12^♀−/♂+^* embryos RNA-seq results.**

| Sample | Total reads | Uniquely mapping efficiency | Mapping efficiency |
| --- | --- | --- | --- |
| gGO_Tcf12_Ctrl_rep1 | 19,019,004 | 35.39% | 60.44% |
| gGO_Tcf12_Ctrl_rep2 | 20,760,101 | 34.78% | 58.91% |
| gGO_Tcf12_Ctrl_rep3 | 22,711,989 | 36.57% | 60.93% |
| gGO_Tcf12_cKO_rep1 | 21,174,961 | 36.12% | 59.96% |
| gGO_Tcf12_cKO_rep2 | 20,235,573 | 10.66% | 29.81% |
| gGO_Tcf12_cKO_rep3 | 20,524,780 | 37.98% | 62.99% |
| fGO_Tcf12_Ctrl_rep1 | 19,166,252 | 36.29% | 61.14% |
| fGO_Tcf12_Ctrl_rep2 | 16,008,066 | 32.57% | 58.44% |
| fGO_Tcf12_Ctrl_rep3 | 18,793,767 | 34.64% | 58.79% |
| fGO_Tcf12_cKO_rep1 | 16,528,903 | 35.07% | 58.81% |
| fGO_Tcf12_cKO_rep2 | 19,645,886 | 36.27% | 60.85% |
| fGO_Tcf12_cKO_rep3 | 18,652,387 | 33.38% | 58.19% |
| 2cell_Tcf12_Ctrl_rep1 | 21,511,411 | 27.13% | 49.83% |
| 2cell_Tcf12_Ctrl_rep2 | 23,004,469 | 36.85% | 60.98% |
| 2cell_Tcf12_Ctrl_rep3 | 25,235,127 | 35.74% | 59.59% |
| 2cell_Tcf12_cKO_rep1 | 21,279,966 | 29.48% | 52.75% |
| 2cell_Tcf12_cKO_rep2 | 17,493,619 | 10.10% | 28.13% |
| 2cell_Tcf12_cKO_rep3 | 23,004,723 | 33.51% | 58.05% |
| 4cell_Tcf12_Ctrl_rep1 | 22,003,374 | 37.49% | 59.85% |
| 4cell_Tcf12_Ctrl_rep2 | 22,151,355 | 33.68% | 55.60% |
| 4cell_Tcf12_Ctrl_rep3 | 19,570,298 | 35.67% | 58.25% |
| 4cell_Tcf12_cKO_rep1 | 18,679,521 | 30.94% | 52.36% |
| 4cell_Tcf12_cKO_rep2 | 23,441,095 | 34.02% | 56.40% |
| 4cell_Tcf12_cKO_rep3 | 22,313,226 | 36.62% | 57.91% |

**Table S5. FPKMs of RNA-seq results (in a separate xlsx file).**

**Table S6. FPKMs of transcripts decreased or increased for more than 2 folds in *Tcf12*^−/−^ oocyte or *Tcf12^♀−/♂+^* embryo samples (in a separate xlsx file).**

**Table S7. Detected proteins of LC-MS/MS analysis (in a separate xlsx file).**

**Table S8. Detected proteins decreased or increased for more than 2 folds in *Tcf12*^−/−^ oocyte samples (in a separate xlsx file).**
